# Keap1–MCM3 interaction is a potential coordinator of molecular machineries of antioxidant response and genomic DNA replication in metazoa

**DOI:** 10.1038/s41598-018-30562-y

**Published:** 2018-08-14

**Authors:** Nele Tamberg, Siret Tahk, Sandra Koit, Kersti Kristjuhan, Sergo Kasvandik, Arnold Kristjuhan, Ivar Ilves

**Affiliations:** 1 Institute of Technology, University of Tartu, Tartu, 50411 Estonia; 2 Institute of Molecular and Cell Biology, University of Tartu, Tartu, 51010 Estonia

## Abstract

Coordination of DNA replication and cellular redox homeostasis mechanisms is essential for the sustained genome stability due to the sensitivity of replicating DNA to oxidation. However, substantial gaps remain in our knowledge of underlying molecular pathways. In this study, we characterise the interaction of Keap1, a central antioxidant response regulator in Metazoa, with the replicative helicase subunit protein MCM3. Our analysis suggests that structural determinants of the interaction of Keap1 with its critical downstream target - Nrf2 master transactivator of oxidative stress response genes – may have evolved in evolution to mimic the conserved helix-2-insert motif of MCM3. We show that this has led to a competition between MCM3 and Nrf2 proteins for Keap1 binding, and likely recruited MCM3 for the competitive binding dependent modulation of Keap1 controlled Nrf2 activities. We hypothesise that such mechanism could help to adjust the Keap1-Nrf2 antioxidant response pathway according to the proliferative and replicative status of the cell, with possible reciprocal implications also for the regulation of cellular functions of MCM3. Altogether this suggests about important role of Keap1-MCM3 interaction in the cross-talk between replisome and redox homeostasis machineries in metazoan cells.

## Introduction

Precise replication of genomic DNA before each cell division is essential for maintaining the integrity of genetic information in proliferating cells and through succession of generations. This process is highly coordinated and monitored by a complex quality control network, which also counteracts genotoxic effects of various stress conditions. One of the central targets of these regulatory pathways is a Cdc45-MCM2-7-GINS (CMG) replicative helicase complex that unwinds genomic DNA in front of the progressing replisome^1–4^. The molecular motor of CMG, formed by a ring-shaped MCM2-7 heterohexamer, is loaded on double stranded DNA already in the G1 phase of the cell cycle^5,6^, but activated as an helicase only in the S phase by assisted recruitment of Cdc45 and GINS accessory subunits^7,8^. These steps determine proper timing and initiation sites of the genomic DNA replication. Also the correct completion of the genome replication relies on active disassembly of CMG complexes on terminating replication forks^9,10^. Genome replication is tightly coordinated with other cellular processes and its proper execution requires the cellular environment to be adjusted according to the specific needs of DNA replication machinery.

Another important aspect of the cellular homeostasis involves the maintenance of intracellular redox balance. Physiological levels of oxidants, such as reactive oxygen species, are generated as by-products of aerobic metabolism and messenger molecules in redox signalling pathways. However, chronic high levels of intracellular oxidants or reactive xenobiotics can overwhelm the cell and induce DNA lesions, accumulation of damaged biomolecules, and development of several associated pathologies like neurodegeneration, aging, and cancer^11^. The expression of many detoxifying genes that counteract these harmful effects is switched on by the transcription activator Nrf2, one of the master regulators of cellular antioxidant response. Nrf2 protein is rapidly degraded in normal cells by 26S proteasome. This is driven by the polyubiquitination of Nrf2, induced by E3 ubiquitin ligase specificity factor Keap1^12–15^ and requiring simultaneous interaction of one Keap1 dimer with the separate high and low affinity beta hairpins of the same Nrf2 molecule^16–18^. In conditions of oxidative or electrophilic stress, such ubiquitination dependent degradation is disrupted and Nrf2 stabilised as a result of poorly understood structural changes in Keap1 protein, which take place after modifications of several specific sensory cysteines in Keap1^15,19–21^. Both the high and low affinity beta hairpins of Nrf2 interact structurally in a very similar manner with the same shallow binding pocket in the Kelch domain of Keap1. The high affinity interaction is determined by the residues of conserved DxETGE loop at the turn of respective beta hairpin of Nrf2^22–24^. This DxETGE interaction motif as well as the structural principles of its interaction with Keap1 are conserved amongst a subset of Keap1 partners^25–27^. Keap1-Nrf2 interaction surface is frequently affected by mutations in cancers, underscoring critical role of the associated pathway in cell physiology and homeostasis, and suggesting about its specific targeting during cancerogenesis^28^.

Here we independently confirm that Keap1 is an abundant binding partner of replicative helicase subunit protein MCM3 in mammalian cells^25,29^. We show that structural principles of the Keap1-Nrf2 interaction have evolved in evolution to mimic the highly conserved helix-2-insert (H2I) motif of MCM3. This has led to the competition between MCM3 and Nrf2 proteins for Keap1 binding, likely recruiting MCM3 for the competitive binding dependent modulation of Keap1-Nrf2 antioxidant response pathway. We propose that such competitive binding mechanism may have enabled the Keap1-Nrf2 pathway to adjust to the status of replication machinery in the cell; the levels of MCM3 competitor, or its availability for Keap1 binding, serving as an indicator of such status. This prototype MCM3 dependent modulation mechanism of Keap1 controlled cellular functions might have further evolved to incorporate similar competitive binding dependent sensory feedback from other proteins and cellular processes^30,31^, possibly enabling precise tuning of the Keap1 controlled regulatory network in response to a wide range of cellular conditions. Our data also suggest about possible involvement of MCM7, another subunit of MCM2-7 complex, and MCM-BP, a protein that can dissociate and unload MCM2-7 complexes from chromatin^32–34^, in the Keap1-MCM3 interaction related regulatory pathways.

## Results

### Keap1 is an interaction partner of MCM3

**Figure 1:**
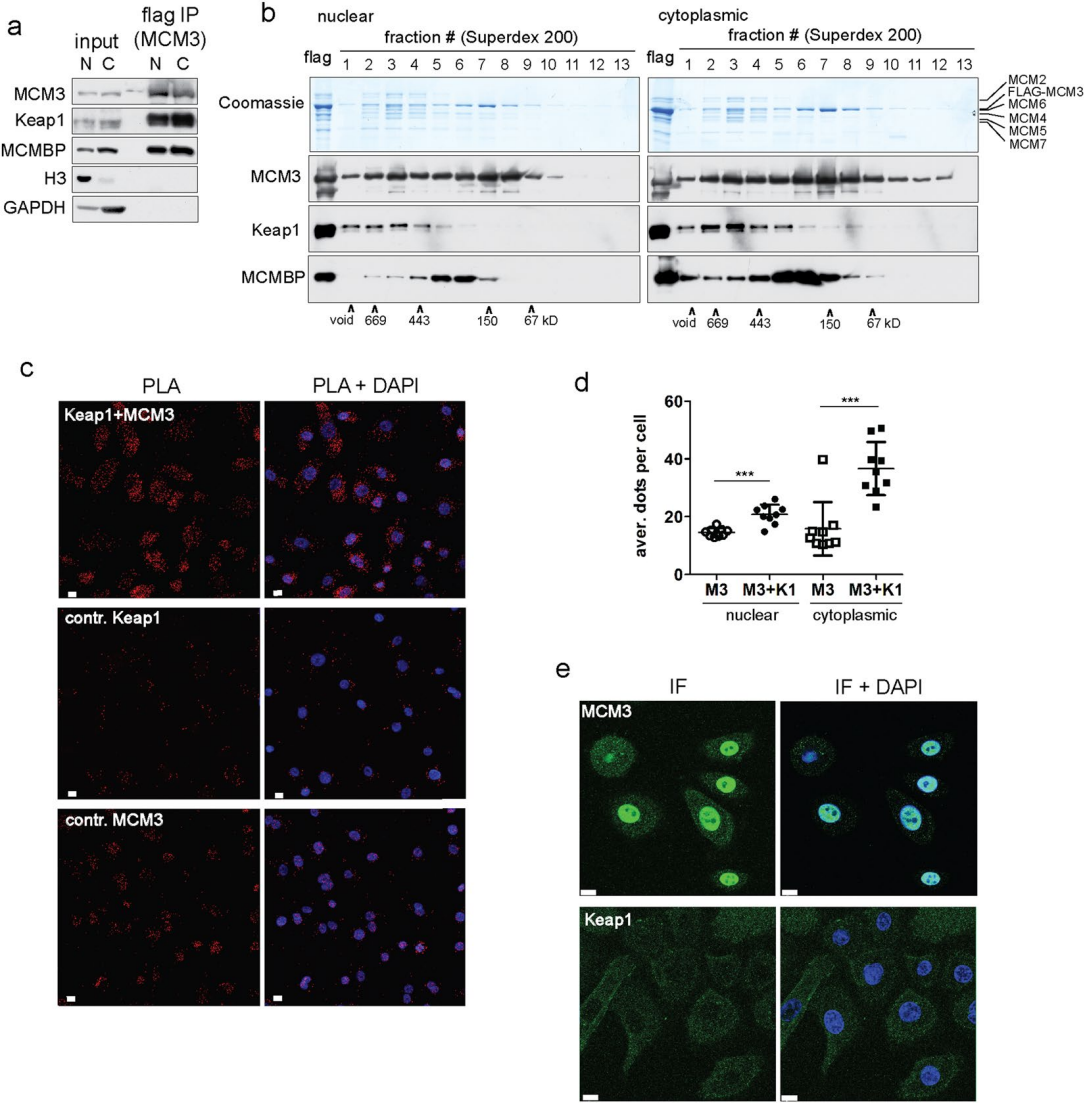
Keap1 interacts with MCM3 in mammalian cells. (**a**) Western blots with antibodies against indicated proteins either with nuclear (‘N’) or cytoplasmic (‘C’) extracts of the FLAG-MCM3 expressing CHO-EBNALT85 cells (‘input’), or in MCM3 complexes immunoprecipitated with anti-FLAG affinity beads (‘flag IP’). Histone H3 and GAPDH were used as fractionation controls. See Supplementary Fig. S2a for full-length blots. (**b**) Coomassie brilliant blue stained SDS-PAGE gels (top panels) and Western blots with antibodies against indicated proteins (bottom panels) showing distribution of FLAG-MCM3 immunoprecipitated nuclear and cytoplasmic protein complexes in the Superdex 200 size exclusion chromatography. ‘flag’ depicts the lanes with input material. Co-elution of molecular weight markers is indicated at the bottom. See Supplementary Fig. S2b for full-length gels and blots. (**c**) Proximity ligation analysis (PLA) of the Keap1 - MCM3 interaction in human primary epithelial keratinocytes (HPEK). The images of red PLA channel alone are shown in the left column, and combined with blue DAPI staining of nuclei in the right column. ‘Keap1 + MCM3’ indicates the images with interaction specific signals, other images correspond to the control experiments with single antibodies. Shown are the maximum intensity projection images of the Z stacks from confocal microscopy; white scale bar = 10 µM. (**d**) Scatter dot plot of the quantified data of nuclear and cytoplasmic Keap1 + MCM3 PLA signals (M3 + K1) compared to negative control with MCM3 antibody alone (M3). Each data point represents an average number of nuclear or cytoplasmic PLA dots per cell from one micrograph. Bars represent the mean and standard deviation of combined data from two independent PLA experiments, one slide analysed in first and two in second experiment and three different micrographs quantified from each slide. The significance values (***p < 0.0005) are derived from unpaired two-tailed t test. (**e**) Immunofluorescence images of the overall distribution of MCM3 and Keap1 proteins in HPEK cells. The images from green protein channel alone are in the left column, and combined with the blue nuclear DAPI staining in the right column. White scale bar = 10 µM.

Searching for the novel interactors and potential regulators of the replicative helicase complex, we co-immunoprecipitated Keap1 in soluble MCM3 containing protein assemblies from the extracts of Chinese hamster ovary (CHO) cells ectopically expressing FLAG affinity tagged MCM3. This independently confirmed previous reports about this interaction in human tissue culture cells, which have identified MCM3 as one of the most abundant cellular partners of Keap1^25,29^. The results of our mass spectrometry analysis showed that after excluding the common contaminants and background interactors, the most abundant specific partners of MCM3 were the other subunits of MCM2-7 complex, Keap1, and MCM-BP (Supplementary Fig. S1). MCM3 immunoprecipitated Keap1 from the whole cell extracts as well as separately from nuclear and cytoplasm enriched fractions, and we estimate that as much as 5–10% of soluble MCM3 and Keap1 proteins were pulled down together in these experiments (Figs 1a,b and S1). The proximity ligation analysis (PLA) of human primary epidermal keratinocytes confirmed the Keap1-MCM3 interaction *in vivo* and indicated that it occurs also in primary human cells, between endogenous proteins in their native subcellular context, and at normal cellular levels (Fig. 1c). Quantification of Keap1 - MCM3 proximity specific PLA dots confirmed that the interaction between these two proteins takes place in both nuclear and cytoplasmic compartments, with some preferential bias towards cytoplasm (Fig. 1d), while the parallel immunofluorescence analysis expectedly revealed that MCM3 is mostly nuclear and Keap1 mostly cytoplasmic in these cells (Fig. 1e).

### MCM3 and Nrf2 interact with Keap1 through highly similar structure and sequence elements, competing with each other for Keap1 binding

**Figure 2:**
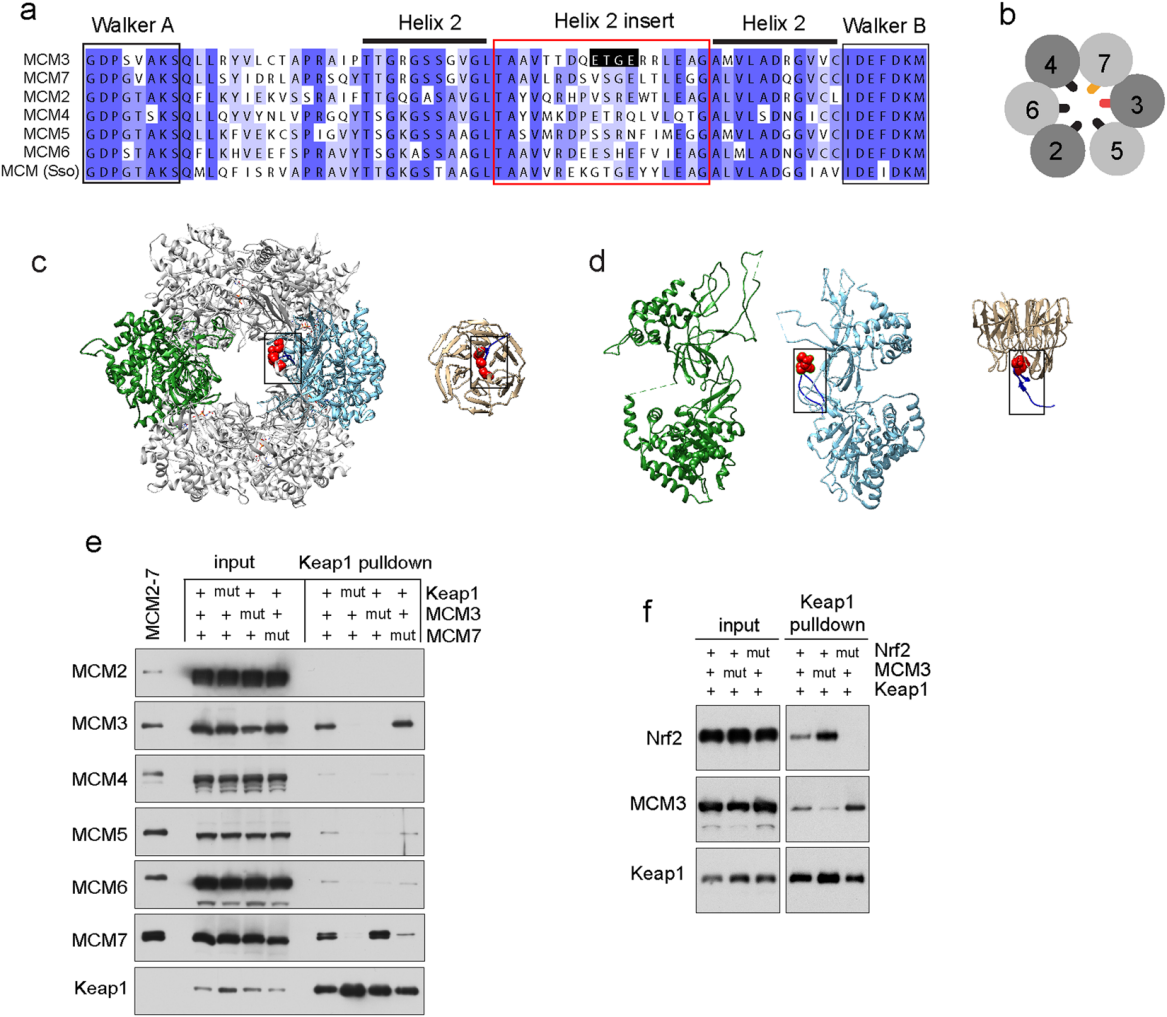
MCM3 and Nrf2 bind to Keap1 in structurally highly similar and competitive manner. (**a**) Sequence alignment of the H2I beta hairpin motifs from human MCM2-7 and *Sulfolobus solfataricus* (Sso) MCM proteins. (**b**) A cartoon showing the conserved order of MCM subunits in MCM2-7 heterohexamer and H2I hairpins in the central channel. (**c**) Structure models of *Saccharomyces cerevisiae* single MCM2-7 complex on the left (PDB accession code 3JA8^38^) and a Kelch domain of human Keap1 bound to DxETGE motif peptide from Nrf2 on the right (PDB accession code 2flu^22^). Kelch domain (beige) is viewed from the side opposite to the binding pocket. MCM2-7 is shown as a top view on its N-terminal tier, MCM3 subunit coloured light blue and opposite MCM6 subunit green. The Keap1 interacting beta hairpin motifs of MCM3 and Nrf2 proteins are in dark blue and marked by boxes here and on panel ‘d’, with ETGE box residues presented by red sphere models. (**d**) Side view (horizontal clockwise 90° rotation) of the same models, where all the other MCM subunits apart from MCM3 and MCM6 have been removed to reveal the central channel of MCM2-7 ring. (**e**) Keap1 pulldown from baculovirus infected Sf9 cells co-expressing all six mouse MCM2-7 proteins and a strep tagged Keap1. Western blots show the protein levels in input extracts (left lanes) and in pulldown samples (right lanes) with co-expressed wt (‘+’) or interaction deficient mutant (‘mut’) proteins as indicated on top. Purified stoichiometric mouse MCM2-7 was loaded on the first lane (‘MCM2-7’) as a reference for comparing different MCM blots. 1/300th of the input extract and 1/6th of the pulldown samples were loaded on each lane. See Supplementary Fig. S4a for images of full-length blots. (**f**) Western blot analysis of Keap1 pulldown experiment from baculovirus co-infected Sf9 cells co-expressing Nrf2 and MCM3 proteins with strep tagged Keap1. Keap1-Nrf2-MCM3 viruses were co-infected at the ratio of 0.1: 0. 5: 3.0 See Supplementary Fig. S4b for images of full-length blots.

The DxETGE high affinity interaction consensus of Keap1 binding proteins, like Nrf2, makes contacts with the residues within a shallow binding pocket formed on top of six C-terminal Kelch repeats of Keap1 positioned in a propeller-like configuration^22–24^. In the MCM3 protein, the identical DxETGE motif is present in the helix-2-insert (H2I) beta hairpin, a defining feature of the MCM clade of AAA+ proteins from archaea to humans (Fig. 2a). The H2I hairpins protrude into a channel formed in the centre of MCM2-7 ring (Fig. 2b–d) and are suspected to be one of the structure elements that make direct contacts with the template DNA strand during the ATP hydrolysis driven DNA unwinding by CMG helicase^35^. Comparison of the 3D structures of MCM2-7 complex from *Saccharomyces cerevisiae* and the DxETGE motif peptide from human Nrf2 in complex with Kelch domain of Keap1 reveals remarkably high structural similarity between the MCM3 H2I and Nrf2 DxETGE hairpins, including the positioning of DxETGE residues at the turn of both beta hairpins (Fig. 2c,d). Although we have used the structure data of yeast MCM2-7 here as no high resolution structure is currently available for MCM2-7 from mammals, the structure of this complex is expected to be highly similar in all eukaryotes. For instance, the structural features of yeast and fruit fly MCM2-7 alone or in CMG complex are remarkably similar (Supplementary Fig. S3), and the crystal structures of human GINS and Cdc45 dock perfectly into the structure model of fruit fly CMG^36^.

We showed the involvement of MCM3 H2I in the interaction with Keap1 in the pulldown experiments from the extracts of Sf9 cells co-infected with baculoviruses expressing all six mouse MCM2-7 subunit proteins together with the strep affinity tagged Keap1. T to A substitution in the DxETGE motif was sufficient to abolish the binding of MCM3 to Keap1 in these assays, as was the R to A substitution of the residues 380 and 415 in the Kelch domain of Keap1 that make specific electrostatic contacts with the glutamate residues within the DxETGE motif of Nrf2 (Fig. 2e)^22,23^. This confirms that the Keap1 - MCM3 interaction is direct^29^ and determined by structural features and residues that closely mimic those used in high affinity Keap1 - Nrf2 interaction. This also suggests that MCM3 and Nrf2 likely compete with each other for the Keap1 binding.

To test this assumption, we carried out similar Keap1 pull-down experiments from the extracts of Sf9 cells, which were co-infected with either WT or Keap1 interaction deficient mutant MCM3 and Nrf2 expressing viruses (Fig. 2f). We found that Keap1 pulled down less Nrf2 as well as MCM3 when both co-expressed proteins were WT compared to when the other co-expressed protein was Keap1 binding defective mutant (Fig. 2f). These data are indeed consistent with the competitive binding of MCM3 and Nrf2 to Keap1, as inferred from the use of highly similar structure elements in both interactions.

### Binding of Keap1 to MCM3 is incompatible with the stable incorporation of MCM3 into full MCM2-7 complex

**Figure 3:**
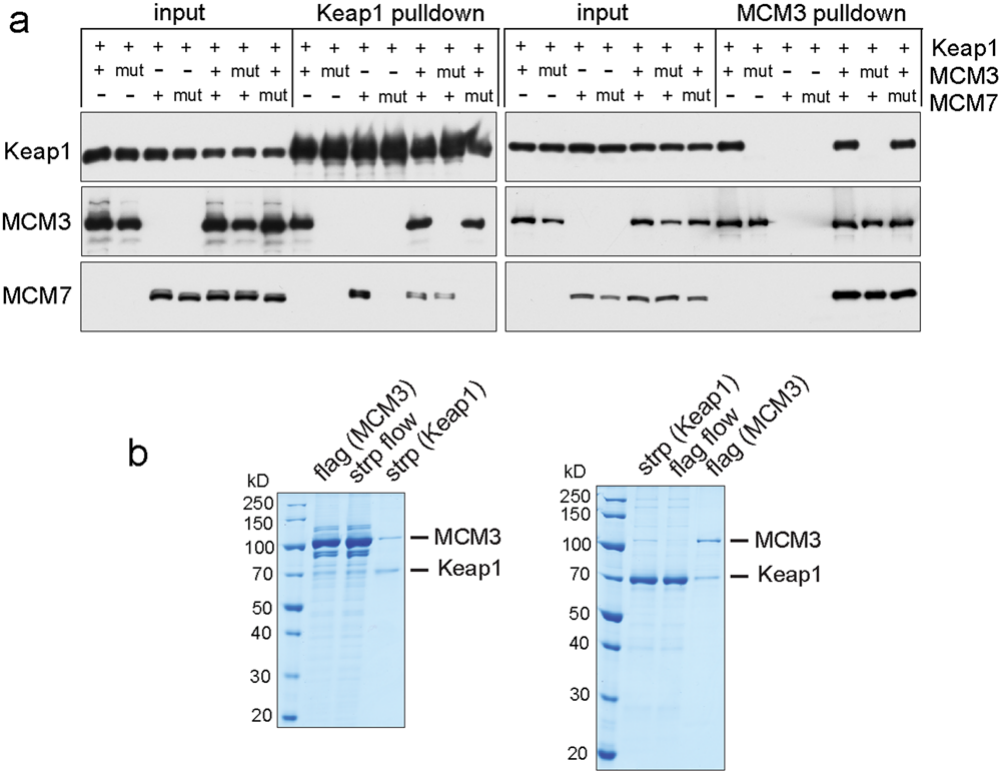
Characterisation of Keap1-MCM3 interaction. (**a**) Strep-Keap1 and FLAG-MCM3 pulldown from the baculovirus infected cells expressing indicated combinations of mouse Keap1, MCM3, and MCM7 proteins. Western blots show the protein levels in input extracts (left lanes) and in pulldown samples (right lanes). WT (‘+’) or interaction deficient mutant (‘mut’) proteins were co-expressed as indicated on top. 1/300th of the input extract and 1/6th of the pulldown samples were loaded on each lane. See Supplementary Fig. S5 for images of full-length blots. (**b**) Coomassie brilliant blue stained SDS-PAGE gels of FLAG-MCM3 – strep-Keap1 tandem affinity pulldown (left panel), and strep-Keap1 – FLAG-MCM3 tandem affinity pull down (right panel) from the baculovirus infected Sf9 cells expressing mouse Keap1 and all six MCM2-7 subunit proteins. Lanes correspond to the eluted material from both pulldown steps and to the unbound material (‘flow’) from the second step as indicated.

We noticed that apart from MCM3, Keap1 was also able to pull down MCM7 in our baculovirus co-expression experiments (Fig. 2e). The H2I beta hairpin of mammalian MCM7 proteins carries DxVSGE motif similar to the DxETGE high affinity interaction consensus (Fig. 2a). The E to A substitution in this motif or R380A/R415A substitutions in Kelch domain of Keap1 abolished the pulldown of MCM7 by Keap1, verifying the participation of these structure elements in interaction (Figs 2e and 3a).

However, although MCM3 and MCM7 are neighbouring subunits in the MCM2-7 hexamer and thus interact as well, we did not detect any stable ternary complex formation between these two and a Keap1 dimer. In fact, the pulldown of MCM7 by Keap1 was decreased by the co-expression of both the wild-type (WT) as well as Keap1 interaction defective ETGE > EAGE mutant MCM3, suggesting about the interfering effect of MCM3 binding to MCM7 on the simultaneous binding of MCM7 to Keap1 (Fig. 3a). Furthermore, although the MCM2-7 proteins are known to efficiently form full heterohexamers and its sub-assemblies, there was very little or no WT MCM3 mediated pulldown of mutant MCM7 or any other MCM subunits by Keap1 in described co-expression experiments (Figs 2e and 3a). MCM3 was the only MCM subunit significantly present in the final complexes with Keap1 also in the MCM3 - Keap1 or Keap1 - MCM3 tandem pulldown experiments from the Sf9 cells co-expressing Keap1 with all six MCM subunits (Fig. 3b), despite the efficient incorporation of MCM3 into the full or partial MCM2-7 assemblies in baculovirus co-expression experiments (Supplementary Fig. S7). Hence, Keap1 binding is either limited to the monomeric MCM3 molecules, for example before being incorporated into MCM2-7 complexes after translation, or the binding of Keap1 could actively contribute to the dissociation of targeted MCM3 from other subunits of MCM2-7 complex, or both. In support of this conclusion, very low levels of MCM2-7 subunits other than MCM3 were immunoprecipitated by Keap1 from human HEK293T cells according to the normalized spectral abundance factor (NSAF) quantification^25^. In another independent proteomic study, no co-immunoprecipitation of Keap1 by MCM2 was detected from human U2OS cells, despite the efficient pull-down of all the other MCM2-7 subunits by MCM2 in these experiments, including the Keap1 interacting MCM3^37^. We thus conclude that although Keap1 can additionally bind to MCM7, MCM3 is its primary target from the MCM2-7 subunit proteins. In addition, and contrary to the previous suggestions^29^, the binding of Keap1 is incompatible with the stable incorporation of MCM3 into MCM2-7 complexes. As the assembly and activity of the replicative helicase complex depend on the full MCM2-7 heterohexamer, the binding of Keap1 to MCM3 subunit likely interferes with these functions as well.

Possible explanation to the destabilizing effect of Keap1 interaction on MCM2-7 is provided by the available protein structure information. The Keap1 interacting H2I hairpin of MCM3 protrudes into central channel of MCM2-7 ring, the general diameter of which, according to the budding yeast structure model with currently the highest available resolution, is 30–40 Å, with even narrower constriction point formed by the H2I hairpins of all six MCM subunits^38^. Similar diameter characterizes the central channel also in lower resolution models of fruit fly MCM2-7^39^ and more distantly related archaeal MCM homohexamer^40^. As the Kelch domain of Keap1 is ~50 Å in diameter and ~35 Å in height^22,23,41^, its binding to the H2I of MCM3 may be sterically hindered in the central channel of the MCM2-7 hexamer, requiring either disassembly or significant and probably destabilizing distortion of the MCM2-7 ring (Figs 2c,d and S3).

### MCM-BP forms a ternary complex with MCM3 and Keap1, suggesting about related regulatory pathway

We noticed that according to the size exclusion chromatography, most of the MCM3 complexes with Keap1 and part of its complexes with MCM-BP that we immunoprecipitated from CHO cells have molecular weight in the range of 400–500 kD or higher (Figs 1b and S1a,c). This indicated that MCM-BP and Keap1 proteins (estimated molecular weights 74 kD and 71 kD, respectively) can both participate in some stable protein assemblies that are larger than just the simple heterodimers with MCM3 (92 kD). As both the Keap1 and MCM3 have been previously isolated from cells in the same protein complexes with MCM-BP^25,33^, we decided to test if MCM3, MCM-BP, and Keap1 can directly form a ternary complex.

**Figure 4:**
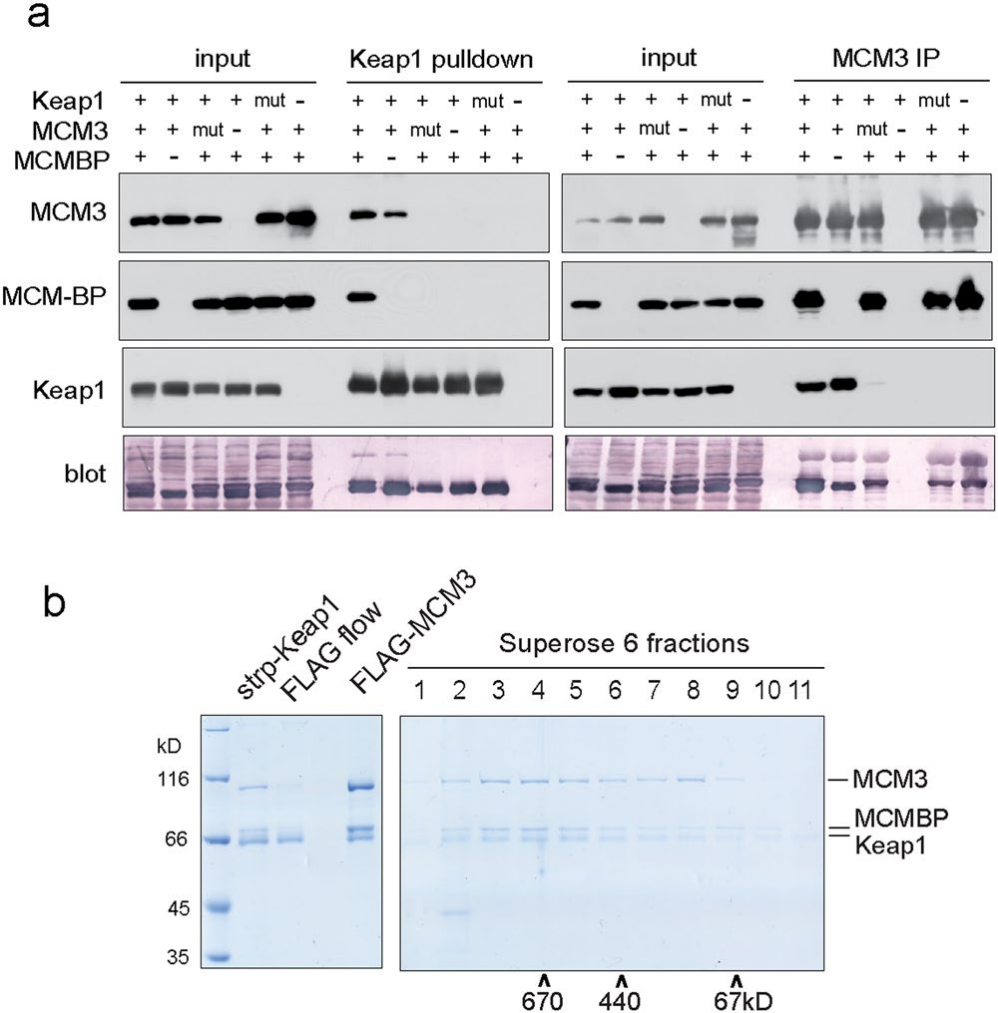
Keap1, MCM3, and MCM-BP form a ternary complex. (**a**) Strep-Keap1 and FLAG-MCM3 pulldown experiments from Sf9 cells co-infected with baculoviruses expressing mouse MCM-BP together with WT or interaction deficient mutant MCM3 and Keap1 as indicated. Top panels show the Western blots of indicated proteins, bottom panel the blotted membranes that were stained with colloidal gold total protein stain. 1/300th of the starting extracts (‘input’) and 1/6th of the pulldown samples was loaded on each lane. See Supplementary Fig. S6 for full-length blots. (**b**) Strep-Keap1 - FLAG-MCM3 tandem affinity purification experiment from Sf9 cells co-infected with baculoviruses expressing all six mouse MCM2-7 subunits, Keap1, and MCM-BP. Coomassie brilliant blue stained SDS-PAGE gel on the left shows eluted material from both affinity purification steps, and unbound material from the FLAG affinity step in the middle lane. Resulting complexes were further resolved by Superose 6 size exclusion chromatography, the fractions of which are shown on right gel; co-elution of molecular weight markers is indicated at the bottom. The identity of protein bands was verified by mass spectrometry.

To accomplish this, we carried out pulldown assays from the Sf9 cells over-expressing these proteins from respective baculoviruses. In these experiments, MCM3 immunoprecipitated MCM-BP and Keap1 both separately as well as when all three were co-expressed (Fig. 4a). The pulldown of MCM-BP by MCM3 was unaffected by mutations that abolished the binding of MCM3 to Keap1. On the other hand, Keap1 pulled down MCM-BP only when MCM3 was additionally co-expressed, and when both Keap1 and MCM3 were WT and able to bind each other (Fig. 4a). These results show that MCM-BP, MCM3, and Keap1 can indeed form a ternary complex, where otherwise non-interacting MCM-BP and Keap1 proteins are brought together by the simultaneous binding to the separate surfaces of the same MCM3 molecule. We were able to isolate this stable Keap1 - MCM3 - MCM-BP complex also by Keap1 - MCM3 tandem affinity pulldown from Sf9 cells co-expressing Keap1 and MCM-BP together with all six MCM2-7 proteins (Fig. 4b). Supporting our previous conclusions that Keap1 binding excludes the stable association of MCM3 with other subunits of MCM2-7, again only trace amounts of MCM subunits other than MCM3 were present in the final purified complex. Subsequent size exclusion chromatography showed that most of the Keap1, MCM3, and MCM-BP in these complexes co-eluted from the column approximately with the 670 kD molecular weight standard and roughly at 1:1:1 molar stoichiometry according to the densitometry analysis of the coomassie brilliant blue stained SDS-PAGE protein gel. This would be consistent with an hexameric complex, where two heterotrimers of Keap1, MCM3, and MCM-BP are brought together by the dimerization of Keap1 (Fig. 4b; see also Supplementary Fig. S7 for the size exclusion chromatography profiles of Keap1, MCM-BP, and MCM2-7 alone). Near equimolar amounts of MCM3 and MCM-BP were pulled down in the first, Keap1 pulldown step (Fig. 4b, lane ‘strp-Keap1’). This shows high efficiency of ternary complex formation in the baculovirus infected cell extracts, as MCM-BP can be pulled down by Keap1 only through MCM3 (Fig. 4a). In more natural cellular context, previous proteomic study has showed the immunoprecipitation of similar amounts of MCM3 and MCM-BP by Keap1 from human tissue culture cells according to the NSAF quantification data^25^. In our experiments with CHO cells, most of the Keap1 protein that was immunoprecipitated with MCM3 was found in the complexes the estimated molecular weight of which according to the size exclusion chromatography would be consistent with at least monomeric and possibly dimeric Keap1 - MCM3 - MCM-BP heterotrimers (Fig. 1b).

Collectively, these data show efficient recruitment of MCM-BP to the Keap1-MCM3 complexes and suggest about possible involvement of MCM-BP also in cellular pathways related to the Keap1-MCM3 interaction. It could be speculated, for example, that the previously described active disassembly of MCM2-7 complexes by MCM-BP^32,42^ may perhaps facilitate the access of Keap1 to the H2I of MCM3 that is otherwise buried inside the MCM2-7 ring.

### DxETGE motif that mediates the high affinity interaction with Keap1 has emerged in MCM3 before similar motifs in Nrf2 and other Keap1 binding partners

In order to compare the evolutionary history of the DxETGE box containing Keap1 interaction motif in MCM3 and Nrf2 proteins, we explored its relative conservation in orthologues of respective genes. The species list for this analysis was compiled with the aim of sampling broadly across all the main super-groups of consensus eukaryotic phylogeny tree^43^, with more detailed emphasis on Metazoa. In addition to Nrf2, we also examined its closely related paralogue Nrf1. These two Nrf proteins have diverged functionally, but share high sequence similarity and the ability to bind Keap1, thus likely originating from the same ancestral gene^44^.

**Figure 5:**
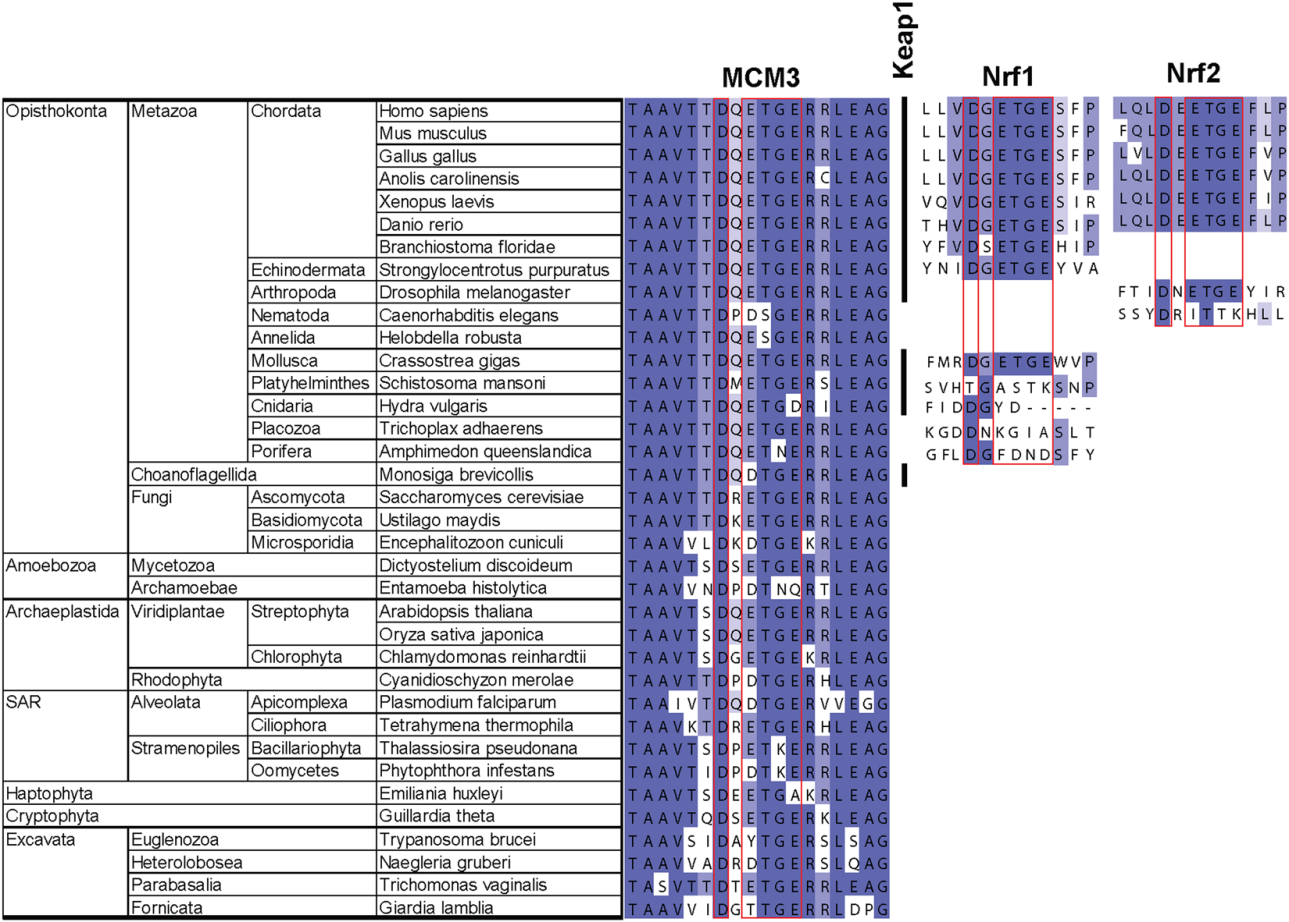
Comparative evolutionary sequence analysis of the DxETGE interaction box in MCM3, Nrf2, and Nrf1 proteins. Sequence homology alignment of DxETGE interaction box and its beta hairpin context in the proteins from indicated species. Black vertical line between MCM3 and Nrf1 columns indicates the presence of Keap1 orthologue in the respective species.

Consistent with the previous studies^44–46^, this analysis showed that genes closely related to human Nrf2 have emerged relatively late in eukaryote evolution, becoming common only in Metazoa (Fig. 5). This timing roughly correlates with the estimated rise in the levels of oxygen on Earth and has thus been suggested to reflect the recruitment of Keap1 into the enhanced control of cytoprotective gene expression by early Nrf2 proteins as an adaptation to such environmental changes^45^. Consistent with this, the presence of a DxETGE consensus box in the Nrf1 and Nrf2 orthologues correlates with the presence of a Keap1 orthologue as its potential binding target in the same species (Fig. 5). Likewise, the residues in Kelch domain that make direct contacts with the DxETGE beta hairpin are well conserved in Keap1 proteins. This agrees with the conserved involvement of mentioned structure elements and residues in the Keap1 - Nrf2 interaction, backed also by the biochemical interaction data with the orthologous proteins from zebrafish and fruit fly^44^.

**Figure 6:**
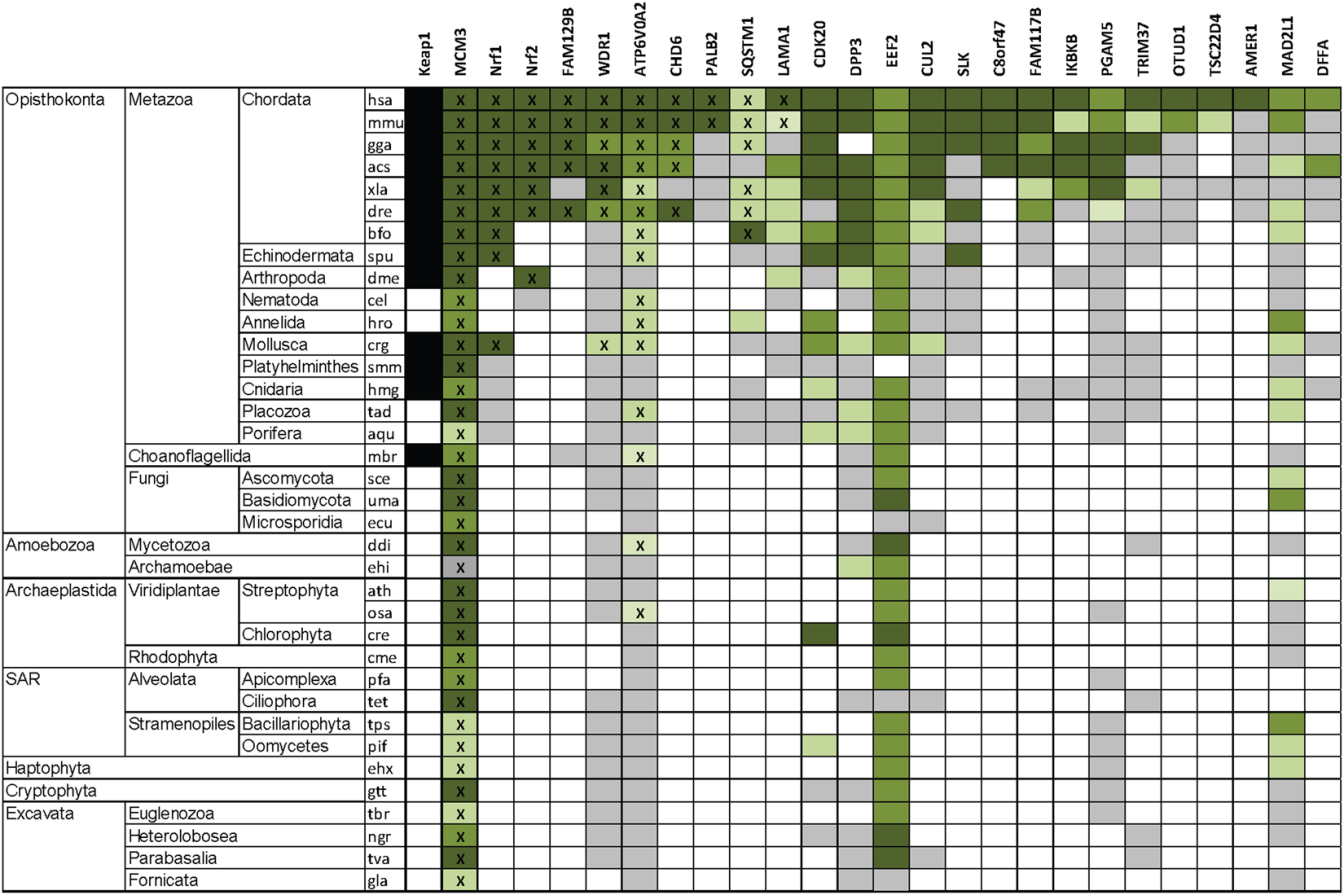
The presence of DxETGE or similar sequence box in the orthologues of characterized or known candidate interaction partners of human Keap1. Comparative evolutionary sequence analysis of the orthologues of identified and candidate partners of human Keap1 that contain ETGE or ESGE consensus motif, or similar DxSTGE motif in case of known Keap1 partner SQSTM1. The conservation is presented using following legend: dark green - ETGE in conserved position; medium green – T > S in human protein, or no more than two conservative E > D or T > S substitutions in other species; light green - one substitution of any other kind plus no more than one additional E > D or T > S substitution; ‘X’ indicates conserved D in -2 position. Grey boxes indicate orthologues with no or very little ETGE similarity, and black boxes in the first column the presence of a Keap1 orthologue. The species are indicated with KEGG organism codes and are listed in the same order as in Fig. 5.

On the other hand, MCM3 gene is present in all eukaryotes and its H2I beta hairpin is highly conserved, as is the DxETGE sequence box in a conserved position at the turn of the hairpin (Fig. 5). The DxETGE box containing beta hairpin motif has thus emerged in MCM3 earlier than in Nrf1 and Nrf2 proteins, also preceding the emergence of a Keap1 protein with the capacity to interact with such motif. In fact, this motif has emerged earlier in MCM3 than in any other Keap1 binding protein. The landscape of Keap1 interaction partners has been exhaustively mapped in human cells and more than 20 proteins that carry ETGE or ESGE motifs have been identified in two independent proteomic screens as well as other studies^25,47,48^. The involvement of these motifs in the direct interaction with Keap1 has been experimentally verified in case of several, albeit not all, proteins in this list. Our comparative sequence analysis indicated that amongst these verified or candidate partners of Keap1, the perfect DxETGE interaction box has appeared the earliest in the orthologues of MCM3, and in other proteins it has emerged approximately about the same time or later in evolution than in the orthologues of Nrf1 and Nrf2 (Fig. 6). Similar sequence motif is relatively well conserved also in EEF2, but this abundant cellular protein is probably a co-purifying contaminant, the direct interaction of which with Keap1 has not been confirmed. The H2I motif of MCM3 is thus evolutionarily older than Keap1 and the regulatory pathways that depend on interactions of Keap1 with this and similar motifs in other proteins, including the Keap1 - Nrf2 cytoprotective pathway.

These observations strongly suggest that the structural principles of Keap1 interaction with Nrf2 and other partner proteins with similar binding mode have evolved to mimic the evolutionarily older H2I motif of MCM3. This also implies that some, if not all, molecular pathways that have emerged through such interactions of Keap1 may have certain mechanistic or regulatory component that incorporates MCM3.

### H2I hairpin of MCM3 together with its conserved DxETGE sequence box is involved in functions of MCM3 critical for cell growth and chromosome maintenance

The conservation of H2I hairpin before the emergence of its interaction with Keap1 suggests that this structure motif is implicated in additional critical functions of MCM3 unrelated to Keap1 binding. Albeit the involvement of H2I motifs in helicase function of MCM2-7 has been suggested by the previous structural studies and by the biochemical analysis of homologous archaeal MCM complex, the role of H2I of MCM3 and specifically its conserved DxETGE motif has previously not been directly addressed^35,49^. Moreover, the MCM2-7 heterohexamer is known to be functionally asymmetric and even the same structure motifs in different subunits of MCM ring can often have different contributions to the functioning of the whole MCM2-7 complex^2,50,51^.

**Figure 7:**
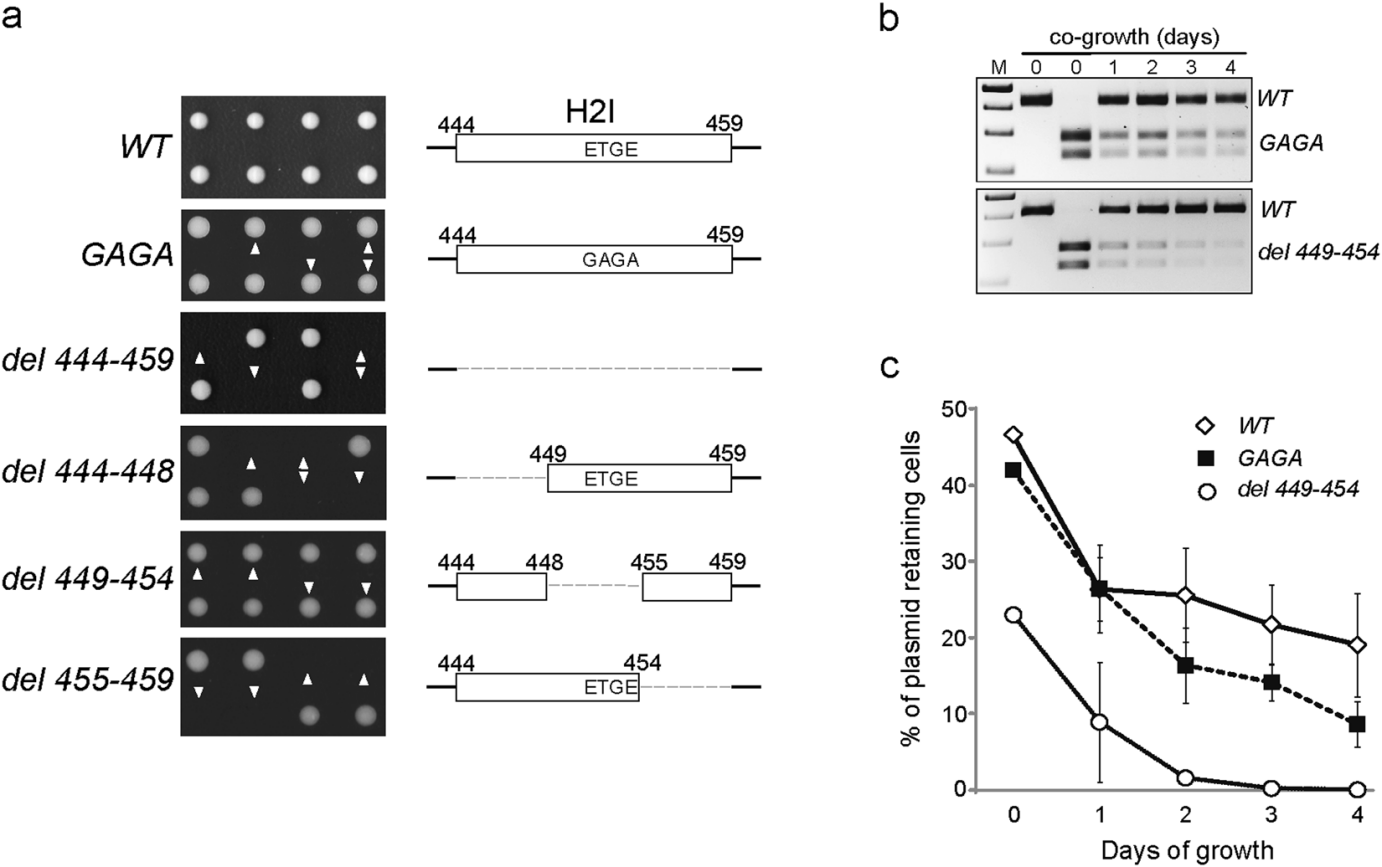
Helix-2 insert (H2I) hairpin and its conserved ETGE sequence box are required for normal growth and minichromosome maintenance function of MCM3 in yeast. (**a**) Tetrad dissection of yeast strains that carry *mcm3* alleles with the mutations in H2I motif as depicted schematically on the right (dashed lines correspond to deleted regions). For each *MCM3/mcm3* diploid strain, two tetrads are shown that were grown for three days after dissection. Arrowheads indicate clones with a mutated allele. (**b**) Competitive co-growth of wild type (WT) yeast with strains carrying *mcm3*-*GAGA* (upper panel) or *mcm3*-*del449*-454 (lower panel) mutant alleles. The WT and mutant strains were pre-grown separately before mixing together on day 0 and co-growing for four days. Genomic DNA from a resulting co-culture was analysed by PCR and following restriction analysis with AluI (*mcm3*-*GAGA*) or XhoI (*mcm3*-*del449*-*454*), which cleave the mutant but not WT DNA fragment. (**c**) Plasmid minichromosome maintenance assay with *mcm3*-*GAGA* and *mcm3*-*del449*-*454* strains. Cells were transformed with pRS416 plasmid and grown for four days without selection, determining the percentage of plasmid-carrying cells each day.

To explore the role of H2I motif of MCM3 and its DxETGE box in more detail, we carried out mutational analysis in budding yeast *Saccharomyces cerevisiae*. Although budding yeast lacks both the Keap1 and Nrf2 proteins as well as the related pathway, the H2I motif in its MCM3 protein differs for example from the H2I of human MCM3 only by one amino acid substitution outside the DxETGE consensus sequence (Fig. 5). We made short deletions in the yeast H2I beta hairpin, or mutated the ETGE sequence box in the genomic copies of MCM3, leaving the overall length of the H2I unchanged (Fig. 7a). All mutations were introduced into one allele of *MCM3* in diploid yeast, followed by sporulation and tetrad dissection of the strains. While deletion of the entire H2I beta hairpin (del444-459) or parts of its “stem” structure (del444-448; del455-459) were lethal and respective clones did not give rise to colonies, the spores carrying either the deletion (del449-454) or GAGA mutation of the ETGE box grew into colonies that were indistinguishable from their WT counterparts (Fig. 7a). To examine the relative fitness of the ETGE box mutants in a more sensitive assay, we co-cultured them with WT *MCM3* strain for four days (approximately 60 generations) and analysed the WT to mutant cell ratio in the culture throughout this period (Fig. 7b). Both tested mutant strains were overgrown by WT strains, although the negative effect of *mcm3-GAGA* mutation on the cell growth was relatively mild compared to the effect of *mcm3-del449-*454 deletion of the ETGE box (Fig. 7b). We also tested these two mutant strains in even more sensitive assay, where the ability of MCM3 protein to support the long term maintenance of an artificial minichromosome DNA with a single replication origin was assessed, unlike in case of genomic DNA replication, which is initiated from multiple origins across each chromosome. We found that both the *mcm3-GAGA* and *mcm3-del449-*454 strains lost the single origin containing minichromosome reporter plasmid pRS416 faster than WT cells over the time in non-selective conditions, with the ETGE truncation again showing stronger negative effect on the growth than ETGE > GAGA mutation (Fig. 7c).

These data show that the H2I beta hairpin of MCM3 together with its DxETGE box are required for normal growth and chromosome maintenance in budding yeast, suggesting the involvement of both in the functions of MCM3 in CMG replicative helicase complex. This has likely provided the selective pressure behind the early conservation of H2I of MCM3 before its interaction with Keap1 emerged. The Keap1 interacting ETGE beta hairpin motif in Nrf2 and other proteins has thus evolved to mimic a functionally important conserved structure motif of MCM3.

### Sensitivity of Keap1 - Nrf2 antioxidant response pathway is modulated by changes in levels of MCM3 protein

If the structural principles of Keap1-Nrf2 interaction have indeed evolved to mimic the H2I motif of MCM3, the immediate question arises about be the mechanistic reason behind this. Supporting the previously reported observations^29^, we did not detect any effect of transient Keap1 overexpression on the overall stability or subcellular localization of the endogenously expressed MCM3 protein (Supplementary Fig. S8a). This indicates that there are probably no general Keap1 dependent effects on the overall cellular MCM3 pool. However, the use of highly similar structure elements by Nrf2 and MCM3 for interacting with Keap1 suggested about the possibility of competitive binding dependent modulation of Keap1-Nrf2 cytoprotective response pathway by MCM3. We thus decided to test if the Keap1-Nrf2 antioxidant stress response is indeed affected by the changes in cellular levels of MCM3 protein.

**Figure 8:**
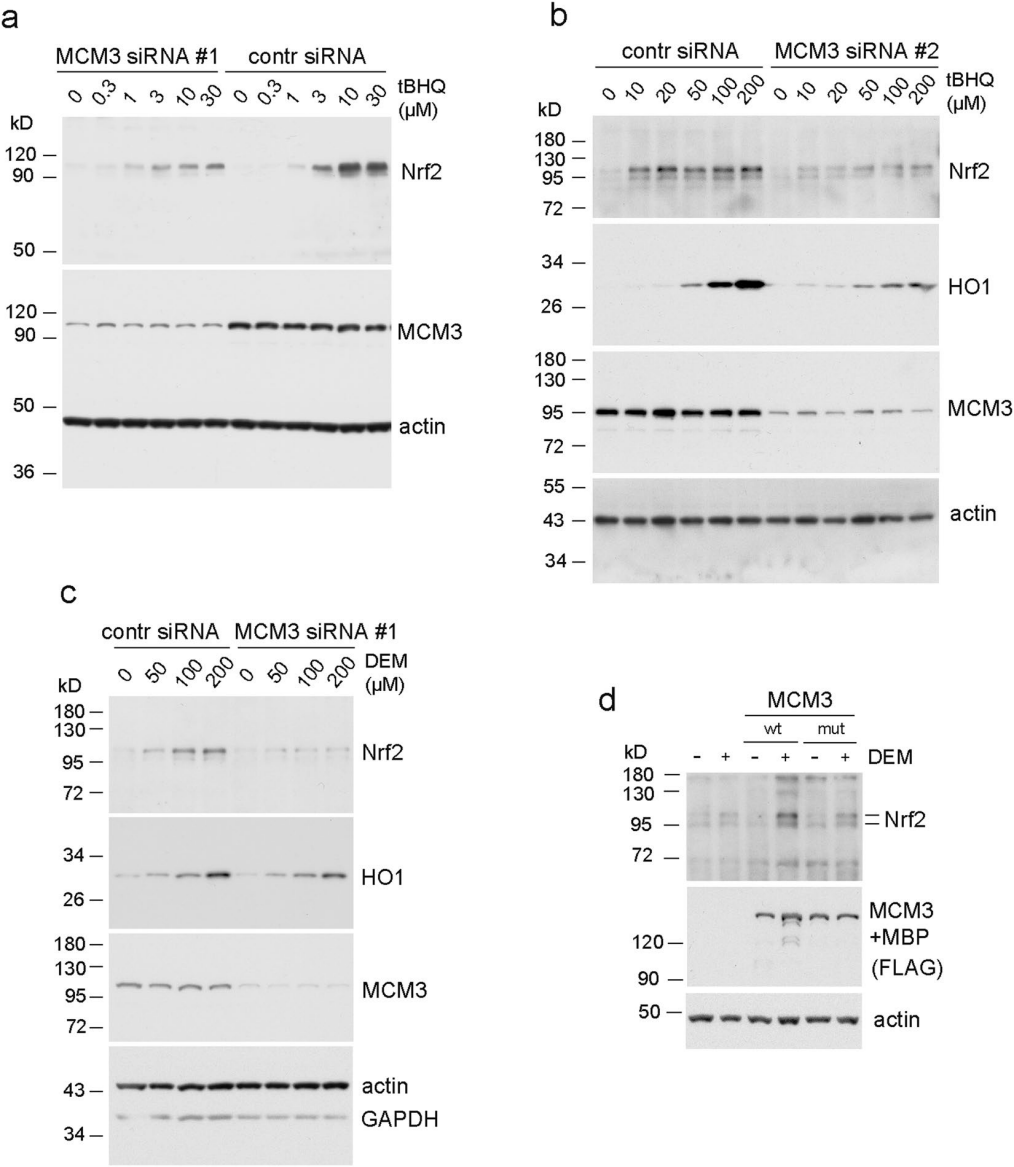
siRNA knock-down of MCM3 levels results in lower sensitivity of Keap1 - Nrf2 response. (**a**) Western blotting analysis of human U2OS cells transfected with MCM3 siRNA #1, or negative control siRNA, and treated with indicated concentrations of tBHQ to induce the Keap1 controlled stabilization of Nrf2 protein. MCM3 blot shows the efficiency of a knock-down and actin blot serves as a loading control in all the panels of this figure. (**b**) Similar experiment, where different siRNA was used (#2) to knock down the MCM3 expression, and cells were treated with higher tBHQ concentrations. Nrf2 transactivation target heme oxygenase 1 (HO1) was additionally blotted. (**c**) The knock-down experiment with MCM3 siRNA #1, where different chemical activator (DEM) was used to induce the Keap1 controlled Nrf2 response. (**d**) Transfection experiments with U2OS cells showing the induction of Nrf2 levels in response to 50 µM DEM treatment (6 hrs) in cells over-expressing either WT or ETGE > GAGA mutant MCM3. Ectopically expressed MCM3 carried N-terminal FLAG and MBP tags and was blotted using antibodies against the FLAG tag of the protein.

To this end, we knocked down the expression of MCM3 protein with RNAi and looked for possible changes in the reaction of Keap1 - Nrf2 pathway to the xenobiotic stress. Such knock-down does not affect the cell growth in the 48 hr time frame of our siRNA experiments^52^, explained by the excess of MCM proteins in the proliferative cells due to the back-up role of most of the cellular MCM2-7 complexes in genome replication^53^. Consistent with this, the MCM3 siRNA transfected cells were growing to equal densities compared to the control cells, as also shown by the similar intensity of actin signal in western blot (Fig. 8a–c). Treatment of human U2OS cells with a known Keap1-Nrf2 pathway activator tert-butylhydroquinone (tBHQ)^54^ induced stabilization of Nrf2 in a concentration dependent manner, as indicated by the higher levels of Nrf2 protein according to the Western blot analysis (Fig. 8a,b). This titration response also showed that the full inhibition of productive Keap1-Nrf2 interaction is not yet achieved at used stress inductor concentrations. Importantly, the induction of Nrf2 required higher concentrations of tBHQ in MCM3 siRNA knock-down cells compared to the control siRNA treated cells. Such effect would be consistent with more efficient destabilisation of Nrf2 in conditions where more Keap1 is available for binding Nrf2 due to lower levels of MCM3 competitor. The result was the same when using different Keap1-Nrf2 pathway activator diethyl maleate^54^ (DEM) (Fig. 8c), and in independent experiments with two different siRNAs (siRNA #1 in Fig. 8a,c; and #2 in Fig. 8b), excluding the possibility of siRNA or inducer specific effects. The lower levels of Nrf2 stabilisation in MCM3 siRNA treated cells were also accompanied with lower levels of induction of the Nrf2 transactivation target heme oxygenase 1 (HO1). The siRNA dependent differences in HO1 induction were observed at higher tBHQ concentrations than required for detecting the differences in Nrf2 stabilisation (Fig. 8b). The effect of siRNA on HO1 induction was rather small in experiments using DEM treatment (Fig. 8c), where higher concentrations of inductor could not be tested due to apparent toxic effect of DEM on cells. Collectively these data show that the Keap1-Nrf2 pathway becomes less sensitive to xenobiotic stress in cells where MCM3 protein levels are downregulated.

We also tested if the ectopic over-expression of MCM3 can activate or sensitise the Keap1-Nrf2 response pathway. To this end, we transfected human U2OS cells with plasmid vectors expressing either WT or Keap1 interaction deficient ETGE > GAGA mutant MCM3. Although the ectopic overexpression of MCM3 alone was insufficient to induce the Nrf2 response in these experimental conditions, the over-expression of WT but not the mutant MCM3 indeed had some enhancing effect on the stabilisation of Nrf2 in DEM treated cells (Fig. 8d). This effect was weak, likely due to the apparent mechanism that seems to control the overall cellular levels of MCM3. We observed much weaker accumulation of MCM3 relative to its endogenous levels in our transfection experiments (Supplementary Fig. S8b) compared to Keap1 (Supplementary Fig. S8a), and major portion of the overexpressed MCM3 was directed to the cytoskeleton enriched fraction (Supplementary Fig. S8b). This means that the achieved levels of MCM3 were probably too low for causing any detectable stabilisation of Nrf2 in non-stressed cells, but sufficient to assert cooperative effect together with the moderate levels of xenobiotic stress.

We conclude from these results that consistent with the competitive binding of MCM3 and Nrf2 to Keap1, the Keap1 dependent activation of Nrf2 cytoprotective response can be modulated by the changes in cellular MCM3 protein levels.

## Discussion

The pathways of redox signalling and oxidative stress response play important and highly complex role in cellular homeostasis. Oxidative signals are often recognised through the oxidation of cysteine residues in specialised sensor proteins, which in turn leads to the changes in conformation, interactions, and activity of these proteins^55,56^. Keap1 is one of the best studied examples of such cysteine sensor proteins due to its role in the control of Nrf2, a master activator of cytoprotective gene transcription in response to oxidative challenges. Apart from the oxidative modifications of its cysteines, the productive interaction of Keap1 with Nrf2 can also be broken by the competitive binding of other proteins like MCM3 that use the same principles of interaction with the Kelch domain of Keap1 as Nrf2^30,31^. Such competition can affect the interaction of Keap1dimer with both the high and low affinity beta hairpins of Nrf2, with several expected outcomes relevant to the activity of Nrf2. Firstly, it could inhibit the Keap1 dependent destabilization of Nrf2 by breaking the interaction of Keap1 dimer with low affinity hairpin of Nrf2. Competitive binding may also fully release Nrf2 from Keap1, especially in oxidizing conditions, where the low affinity hairpin does not contribute to the overall affinity of Keap1dimer to Nrf2. In addition, the bound competitor might inhibit the anchoring of newly translated Nrf2 molecules by Keap1^30,31^.

Notably, our comparative evolutionary analysis revealed that the interaction surface used in such competition mechanism has likely emerged in evolution to mimic the H2I of MCM3. This suggests that MCM3 might have been the first such competitive regulator of Keap1-Nrf2 pathway, emphasizing the primary importance of coordinating the redox homeostasis with genome replication process. More than twenty other proteins have been identified as binding or potentially capable of binding to Keap1 in a manner similar to Nrf2. Additional interactions might thus have taken cues from the initial MCM3 associated mechanism in order to feed the critical information from other cellular processes into the Keap1 - Nrf2 redox homeostasis control pathway. Keap1 might thus have gained a role of a molecular hub that regulates Nrf2 and perhaps some other target proteins by ubiquitination, sequestration, or other means, and directly coordinates this regulation with the DNA replication as well as possibly other cellular processes, such as autophagy^27,57,58^, recombination^59,60^, and perhaps others^25,61–63^.

The observation that the motifs of Nrf2 mediating its interaction with Keap1 have likely emerged to mimic the H2I of MCM3 would be consistent with one of the two possible evolutionary routes. First, that the ancestral Keap1 may have first developed the ability to bind the H2I of MCM3 - perhaps related to some early regulatory function - and similar Keap1 interaction motif later evolved in Nrf2 and other proteins. Alternatively, the Keap1-Nrf2 interaction and related antioxidant response pathway might have emerged first, and the initial interaction motif of Nrf2 later evolved to mimic the DxETGE motif in H2I of MCM3, together with the respective adaptation of the interaction pocket in Keap1. The outcome in both cases would be direct competition between MCM3 and Nrf2 for Keap1 binding. We propose that the driving force behind the emergence of such competition might have been the need for a direct coordination of the Keap1-Nrf2 antioxidant response pathway with the replicative and proliferative status of the cell. Such need is explained by the higher sensitivity of replicating DNA to the oxidizing conditions, which can be counteracted by the higher sensitivity of Nrf2 antioxidant response to the oxidative bursts in the proliferating compared to the non-proliferating cells. MCM2-7 protein expression is a good indicator of proliferative status of the cell, as it is high in proliferating cells and ceases only after the exit from the cell cycle e.g. due to terminal differentiation, quiescence, or senescence^64,65^. The ability of changes in MCM3 levels to modulate the Keap1-Nrf2 response was successfully recapitulated in our MCM3 knock-down and overexpression experiments (Fig. 8).

Apart from the overall levels of MCM3, changes in the accessibility of its H2I hairpin for Keap1 binding may also be implicated in the competitive binding dependent sensitisation of Nrf2 response. This can come about for example as a result of accumulation of non-stoichiometric MCM2-7 complexes or the increased disassembly of MCM2-7 hexamers in replicative stress conditions. In that regard, efficient formation of the MCM-BP - MCM3 - Keap1 ternary complexes (Fig. 4) suggests about one possible mechanism that could directly connect to the Keap1-MCM3 interaction dependent pathways. This comes from the previously characterized involvement of MCM-BP in the chromatin unloading and disassembly of the MCM2-7 hexamers^42^, the exact physiological role of which is still unclear. Future studies on possible links between MCM-BP and the Keap1-MCM3 interaction dependent regulation mechanisms may thus provide clues also about the role of MCM-BP in the cell.

It remains to be shown if Keap1-MCM3 interaction, besides likely acting as a coordinator of Nrf2 antioxidant response with the replicative status of the cells, may also have a direct regulatory role in genomic DNA regulation. The fact that Keap1 knock-out mice do not show any proliferation defects and die postnatally only due to unrelated digestive system defects^66^ indicates that any such potential regulation of MCM3 by Keap1 is probably not essential for the genome replication. However, the importance of coordinating the genome replication with cellular redox status implies that such coordination is likely achieved through the concerted efforts of multiple molecular pathways that can substitute for each other. In yeast, genome replication is coordinated with the periodic metabolic redox fluctuations in a manner that segregates the genomic DNA replication from the potentially genotoxic oxidative phase of the yeast metabolic cycle^67,68^. Such general mechanism is not used in much more complex and sophisticated metazoan cells, although certain redox fluctuations have been reported to coordinate specific S-phase related processes like histone gene expression^69–71^. One mechanism of coping with the intracellular oxidative challenges in metazoa that is linked to the direct regulation of the replication was provided by the recent demonstration that oxidizing conditions can slow down the speed of replication fork. This is caused by the peroxiredoxin 2 linked dissociation of replication fork protection protein TIMELESS from the progressing replisome complexes^72^. Slowing of the replication fork might provide sufficient time for repair of the potential lesions in template DNA, thus avoiding the replicative stress and associated genotoxic effects in conditions of redox fluctuations. Another additional candidate mechanism for directly coordinating the genome replication in metazoa with changes in redox status involves the reported cysteine oxidation dependent regulation of ssDNA binding by RPA, albeit the exact *in vivo* role for such regulatory mechanism remains to be explored^73^. The coordination mechanisms are not limited to the genomic DNA replication, as the oxidation of cysteine residues has been shown to directly regulate the functions of critical replication factors in synchrony with the cellular redox status also in the initiation of kinetoplast DNA replication of trypanosomatids^74,75^, and in the genomic DNA replication of papillomaviruses^76,77^.

Ectopic overexpression of Keap1 does not affect the overall stability or subcellular localization of the endogenous MCM3 protein (Supplementary Fig. S8a)^29^. This suggests about the lack of any robust Keap1 dependent effect on the overall cellular MCM3 pool, although not excluding the possibility of Keap1 perhaps having some role for example in the regulation of specific sub-population of MCM3, or maybe involving specific physiological conditions or cell types. Our observations about the incompatibility of Keap1-MCM3 interaction with the stability of MCM2-7 hexamer suggest that any potential impact of Keap1 on the functions of MCM3 in the replicative helicase component of the replisome could be expected to be inhibitory rather than activatory. Moreover, the binding of Kelch domain of Keap1 to the H2I of MCM3 in the central channel of MCM2-7 ring and the engagement of DNA strand(s) in the same channel are sterically mutually exclusive. This means that any binding of Keap1 to MCM3 even in the intact MCM2-7 hexamer would certainly inhibit the loading of MCM2-7 complex on DNA. The incompatibility of Keap1-MCM3 interaction with the stability of MCM2-7 hexamer could also mean that apart from passively binding to the MCM3 in already disassembled complexes, Keap1 may also have the capacity to actively contribute to the disassembly of MCM2-7, perhaps also in cooperation with MCM-BP. One related speculation could be that Keap1 could sequester the incomplete or disassembled MCM complexes, where the H2I of MCM3 is better accessible for Keap1 binding. This may help to avoid potentially poisonous effects of such complexes on the cell due to the competition with complete MCM2-7 for critical interactions. As the nuclear transport of MCM2-7 complexes has been reported to be controlled by the MCM3 subunit^78–80^, the sequestering of MCM3 containing incomplete assemblies in the cytoplasm would be sufficient to avoid the nuclear transport of any incomplete MCM sub-assemblies. Possibly relevant to this, Keap1 has been proposed to drive the ubiquitination of MCM3^29^. Although the exact cellular role of this modification remains to be shown, it is tempting to speculate that it could mark the incomplete MCM assemblies that are recognised by Keap1 for example for nuclear exclusion or degradation. The interactions of Keap1 with the H2I hairpins of neighbouring MCM3 and MCM7 subunits that we show here present an attractive testable candidate mechanism for the ubiquitination of MCM3, due to obvious parallels with the Keap1 dependent polyubiquitination of Nrf2 that depends on the interaction with both hairpins of Nrf2.

Keap1-MCM3 interaction may thus have possible implications for both the regulation of genome replication as well as redox homeostasis control in the cell, thus providing some of the essential coordination between these two processes.

## Materials and Methods

### Antibodies used in Western blotting

Antibodies against following proteins were used: MCM2 (N19, sc-9839), MCM3 (N19, sc-9850), MCM4 (C-10, sc-48407), MCM5 (33, sc-136366), MCM6 (H-300, sc-22780), MCM6 (C-20, sc-9843), MCM7 (H-5, sc-374403), Heme Oxygenase 1 (HO1) (A-3, sc-136960), actin (I-19, sc-1616), Histone H3 (FL-136, sc-10809), GAPDH (6C5, sc-32233), Lamin B1 (A-11, sc-377000), Sp1 (sc-59), and Keap1 (E-20, sc-15246) - all from Santa Cruz Biotechnology; Keap1 (D6B12), and Nrf2 (D1Z9C) - Cell Signaling Technology; MCM-BP (HPA038481), and anti-FLAG-tag (M2, F1804) - Sigma-Aldrich; anti- basal cell cytokeratin (RCK103, ab9222) - Abcam.

### Isolation of MCM3 containing complexes from tissue culture cells

FLAG-MCM3 expressing CHOEBNALT85 cell lines were constructed by Icosagen Cell Factory, Estonia, using the proprietary QMCF technology^81^. Briefly, the coding region of mouse MCM3 with 5′ in frame FLAG-tag was PCR-cloned into expression vectors pQMCF-1 and pQMCF-6 under the control of CMV or hEF1α promoter, respectively^82^. All the starting cDNA clones used for cloning in this study were obtained from PlasmID Repository at Dana-Farber/Harvard Cancer Center DNA Resource Core, and the coding regions in all completed plasmid constructs were verified by sequencing and alignment against the respective consensus reference sequences in NCBI RefSeqGene database. Resulting FLAG-MCM3 expressing vectors were transfected into Chinese hamster ovary CHO-S - derived cell line CHOEBNALT85, both the pQMCF-1 and pQMCF-6 plasmids yielding very similar expression levels of FLAG-MCM3 protein. Transfected cells were expanded by serial passaging to a desired volume of suspension culture.

For the experiments with whole cell extract (Supplementary Fig. S1a,b), 400 ml of the pQMCF-1-FLAG-MCM3 carrying CHOEBNALT85 culture at density 4.4 × 106 cells/ml was harvested and washed once with PBS (all the steps in this as well as in nuclear/cytoplasmic extract protocol at 4 °C or on ice, unless indicated otherwise). The cell pellet (5 ml) was resuspended in 5 packed cell volumes (25 ml) of hypotonic lysis buffer C-10 (15 mM Hepes-KOH pH 7.8; 10 mM KCl; 2 mM MgCl_2_; 0.05% Tween 20) with supplements (2 mM 2-mercapthoethanol, 0.4 mM PMSF, complete protease inhibitor cocktail without EDTA from Roche) and phosphatase inhibitors (2 mM NaF, 2 mM β-Glycerophosphate). The cell suspension was snap-frozen in liquid nitrogen and stored at −80 °C. Half of the frozen suspension was thawed and homogenized in a Dounce grinder (20 strokes, tight B glass pestle). After incubating on ice for 10 more minutes, the KCl concentration was adjusted to 150 mM, and C-150 buffer with 30% glycerol (15 mM Hepes-KOH pH 7.8; 150 mM KCl; 2 mM MgCl_2_; 0.05% Tween 20; 30% glycerol) was added with supplements and phosphatase inhibitors, to adjust the final concentration of glycerol to 10%. The extracts were cleared 30000 g for 20 minutes and incubated with 100 µl of ANTI-FLAG M2 affinity gel beads (Sigma-Aldrich) on the end-over-end rotator for 4 hours. Beads were collected by centrifugation, resuspended in C-150 buffer with 10% glycerol and transferred to 1.5 ml microcentrifuge tubes before washing 7 times with 1 ml of the same buffer. Proteins were eluted from the beads by incubating for 30 min with continuous end-over-end mixing in 300 µl elution buffer (C-150 + 10% glycerol, 200 µg/ml FLAG peptide, Roche complete protease inhibitor cocktail without EDTA). The eluate was removed after collecting the beads by centrifugation and the elution step was repeated with 200 µl of the elution buffer. The material from the first fraction was injected into Superdex 200 10/300 GL column attached to the GE Healthcare Life Sciences ÄKTAmicro protein purification system. C-150 buffer with 10% glycerol was used as a column buffer, and 750 µl fractions were collected. Proteins from the selected fractions were precipitated with cold 20% TCA, the pellets were washed twice with cold 90% TCA and air-dried before the mass spectrometry analysis.

For the experiments separately with nuclear and cytoplasmic extracts (Figs 1a,b and S1c,d), 400 ml of the pQMCF-6-FLAG-MCM3 carrying CHOEBNALT85 suspension culture grown to density ~8 × 10^6^ cells/ml, or the same amount of non-transfected CHOEBNALT85 control cells, was harvested by centrifugation, washed once with PBS and once with 5 packed cell volumes (25 ml) of hypotonic lysis buffer C-10 (with supplements, but without Tween-20). The phosphatase inhibitors were omitted from all the buffers in this protocol. The cells were resuspended in 3 packed cell volumes (15 ml) of C-10 with supplements and let to swell for 10 min before treating to 12 strokes in Dounce grinder with a tight (B) glass pestle. The efficiency of cell breaking was checked by trypan blue staining and confirmed to be at least 90%. The nuclei were collected by centrifugation at 1900 g and snap-frozen in liquid nitrogen. The supernatant, corresponding to the cytoplasm enriched fraction, was transferred to a fresh tube and the KCl concentration was adjusted to 150 mM; the glycerol concentration was adjusted to 10% by adding C-150 buffer with 30% glycerol and supplements, and the extracts were cleared for 15 min at 30000 rpm before snap-freezing in liquid nitrogen. The frozen nuclear pellets and cytoplasmic extracts were stored at −80 °C. For preparing the extracts, the nuclear pellets (~10 ml) were thawed and resuspended in 1:1 volume (10 ml) of C-10 buffer with supplements and 100 U/ml benzonase. The extracts were incubated for 30 min with end-over-end mixing, treated to 5 strokes with tight pestle (B) in Dounce grinder, and rotated for 20 min more before adjusting KCl concentration to 150 mM. C-150 buffer with 30% glycerol+ supplements was added to adjust the final glycerol concentration to 10%, and the tubes were incubated for additional 30 min with end-over-end mixing. The resulting nuclear extracts and the thawed cytoplasmic extracts were cleared at 30000 g for 20 min before proceeding with the anti-FLAG immunoprecipitation and Superdex 200 chromatography as described above.

### Proteomics sample preparation and nano-LC/MS/MS analysis

Protein pellets were processed into peptides as described previously^83^. Samples were injected to an Agilent 1200 series nano-LC with an in-house packed (3 µm ReproSil-Pur C18AQ particles) 15 cm × 75 µm ID emitter-column (New Objective). Separation was carried out with an 2–40% gradient of buffer B at 200 nl/min for 1 h (buffer A: 0.5% AcOH, buffer B: 80% ACN, 0.5% AcOH) and the peptides were detected (spray voltage 2.0–2.2 kV) with an LTQ Orbitrap XL (Thermo Fisher Scientific) mass-spectrometer (MS). Each MS scan was followed by MS/MS analysis of the 5 most intense peaks. Dynamic exclusion was set to 60 s and only charge states over +1 were analysed. Mass-spectrometric raw data were analyzed with MaxQuant 1.5.3.172^84^. Two missed cleavages were allowed. Carbamidomethylation was set as a fixed modification and methionine oxidation, N-terminal acetylation as variable modifications. Data were searched against RefSeq (https://www.ncbi.nlm.nih.gov/refseq/) *Cricetulus griseus* database (downloaded on 16.03.2017) supplemented with the *Mus musculus* MCM3 sequence. First and main search MS mass tolerances were ≤20 and ≤4.5 ppm, respectively. MS/MS tolerance was ≤20 ppm. Criteria for identification were specified as following: 1 peptide, minimum length of 7 residues and a false discovery rate of <1% using a target decoy approach. Match between runs was enabled. All other parameters were default.

### Baculovirus construction, protein purification, and protein pulldown assays

cDNAs for mouse Keap1, MCM-BP, or MCM2-7 subunit proteins were cloned into the pFastBac1 donor vector and the recombinant baculoviruses were constructed using the Bac-to-Bac protocol and reagents (Thermo Fisher Scientific). FLAG-MCM3, strep-Keap1, and HA-MCM-BP viruses express the proteins containing the N-terminal FLAG, Strep-tag II, or HA affinity tags, respectively. Mutant versions of FLAG-MCM3 and MCM7 carry ETGE > EAGE and VSGE > VSGA substitutions in the helix-2-insert sequences. strep-Keap1 mutant protein contains Arg to Ala substitutions at positions 380 and 415. All the point mutations were introduced into coding regions using standard PCR-based site-directed mutagenesis techniques.

Baculovirus expression and protein purification as well as pull-down experiments were carried out in *Spodoptera frugiperda* Sf9 cells grown in Ex-Cell 420 serum-free media (Sigma-Aldrich). Proteins were expressed for two days after infecting Sf9 cells with baculoviruses at MOI ~5–10. For the small scale co-expression and pull-down experiments, the cells were infected on 15 cm dishes, 2 × 10^7^ cells per dish. The extracts for large-scale protein purification experiments were prepared from the suspension cultures infected at density 10^6^ cells/ml, 300–600 ml for each experiment, using the previously described protocols (2).

In the first step of tandem affinity purification experiments, the cleared extracts were passed twice through either the anti-FLAG M2 affinity gel (Sigma-Aldrich) for capturing the FLAG-MCM3 complexes, or strep-tactin superflow (IBA Lifesciences) for capturing the strep-Keap1 complexes. The columns were washed 3 times with C-100 buffer (100 mM KCl, 25 mM Hepes-KOH pH 7.8, 0.02% Tween-20, 10% glycerol, 0.1 mM EDTA, 0.4 mM PMSF, 2 mM 2-mercaptoethanol). The bound proteins were eluted with 2.5–10 mM desthiobiotin in case of strep-tactin, or 100 µg/ml FLAG peptide in case of anti-FLAG gel, in C-100 buffer supplemented with Roche protease inhibitors cocktail. In the strep-FLAG tandem affinity purification experiments in Fig. 4b, 50 µl of packed anti-FLAG beads, and 250 µg/ml insulin as a crowding agent, was added to the eluate from the first strep-Keap1 step and incubated for 1 hr with continuous end-over-end mixing. Beads were collected by centrifugation and washed 7 times by resuspending in 1 ml 100-C buffer, collecting by centrifugation after each step. Proteins were then eluted by resuspending the beads in 500 µl of 100 µg/ml FLAG peptide in buffer 100-C supplemented with protease inhibitors cocktail and incubating on the end-over-end mixer for 45 min before collecting the eluate. 250 µl of this material was injected into Superose 6 10/300 GL column attached to the GE Healthcare Life Sciences ÄKTAmicro system, using 100-C as a column buffer and collecting 750 µl fractions. In the strep-FLAG and FLAG-strep tandem affinity purification experiments in Fig. 3b, the eluate from the first affinity step was passed 3 times over the column containing 100 µl of the packed second affinity resin, washed 3 times with 10 column volumes of 100-C buffer, and eluted with 3 × 100 µl of elution buffer with 10 mM desthibiotin in case of strep-tactin column, or 250 µg/ml FLAG peptide in case of anti-FLAG column, both in buffer C-100 supplemented with Roche protease inhibitors cocktail. In case of anti-FLAG resin, the column was incubated for 5 min after each addition of the elution buffer before collecting the eluate.

For the FLAG-MCM3 or strep-Keap1 co-expression and pull-down experiments, 10 µl of packed anti-FLAG or 15 µl of packed Strep-Tactin beads was added to the cleared extract from 2 × 10^7^ of infected cells and incubated with end-over-end mixing for 1 hr at 4 °C. The beads were washed four times with buffer C-250 (250 mM KCl, 25 mM Hepes-KOH pH 7.8, 0.02% Tween-20, 10% glycerol, 0.4 mM PMSF, 2 mM 2-mercaptoethanol). Bound proteins were eluted with 30 μL pH 2.5 glycine buffer (50 mM glycine, 150 mM NaCl) and immediately neutralized with 3 μL of 1 M Tris-HCl pH 8.0.

### Proximity ligation assay (PLA) and immunofluorescence (IF) analysis of human primary epithelial keratinocytes

Human primary epidermal keratinocyte progenitor cells (single juvenile donor, passage 2) were purchased from CELLnTEC Advanced Cell Systems and grown for no more than 8 passages in CnT-Prime media provided by the supplier. The cells on coverslips were washed with PBS and fixed in 4% paraformaldehyde for 20 min at room temperature. After three washes with ice cold PBS, the cells were permeabilised for 5 min with 0.1% Triton X-100 in PBS.

The PLA analysis was carried out using the Duolink PLA kit (Sigma-Aldrich) and a protocol provided by the manufacturer. Goat antibody against MCM3 (N19, sc-9850) and mouse antibody against Keap1 (sc-365626; both from Santa Cruz Biotechnology, Inc.) were used as primary probes at 1:50 dilution and incubated at 4 °C overnight. Anti-mouse plus and anti-goat minus PLA probes were used as secondary probes, and the Duolink *In Situ* Detection Reagents Orange reagents were used to amplify and detect the signal. Processed cells were mounted with Duolink *in situ* mounting medium with DAPI and analysed with a Zeiss LSM 710 confocal microscope, collecting Z-stacks consisting of 7–14 confocal layers. ImageJ software^85^ was used to create the maximum intensity projection images from the Z-stacks and for quantification of the nuclear and cytoplasmic PLA dots. For quantification purposes, the nuclear area on each image was defined as stained positive by DAPI and the rest of the PLA dots were considered cytoplasmic, as essentially no signal was present outside the cell borders.

For the IF analysis, the cells were prepared and processed with primary antibodies exactly as in PLA experiments. The samples were then washed with PBS and incubated for 1 hour at the room temperature with the secondary anti-mouse Alexa Fluor 568 and anti-goat Alexa Fluor 488 antibodies (Molecular Probes/Thermo Fisher Scientific) diluted in PBS with 1% BSA and 0.1% Tween 20. The slides were washed in PBS and mounted in the Duolink mounting medium with DAPI before analysis with the confocal microscopy as in case of PLA experiments.

### Protein structure comparison analysis

Molecular graphics and structure comparison analysis were performed with the UCSF Chimera package developed by the Resource for Biocomputing, Visualization, and Informatics at the University of California, San Francisco (supported by NIGMS P41-GM103311)^86^.

### Comparative evolutionary protein sequence alignment analysis

The list of genes for the analysis consisted of the known and candidate interaction partners of human Keap1, which carry either the conserved ETGE or similar interaction box^48^ (and references therein^47^). Functional orthologs for any given gene were identified with the help of Kyoto Encyclopaedia of Genes and Genomes (KEGG, http://www.kegg.jp/)^87^, first searching from the curated KEGG orthology (KO) group containing the respective human gene. For the genes not assigned to any KO group, orthologs were searched from the KEGG Sequence Similarity DataBase (SSDB) best search result list for the respective gene, based on the precomputed pairwise sequence similarity data. The reciprocal best-best hits with Smith-Waterman (SW) score at least 200 were selected. Finally, the unidirectional best hits from the SSDB best search result list for the each analysed human gene were examined, in order to confirm the selection and identify additional candidate orthologues that may have been missed with first two approaches. In case of multiple protein matches from the same organism, the ones carrying ETGE or similar sequence motif in conserved position and/or showing the better precomputed score were selected (weighted KEGG KOALA score in case of KO group, SW-score in case of KEGG SSDB best search result list). Both the curated KO group pages as well as the SSDB search result lists were accessed from the KEGG GENE entry pages for respective human genes. The KEGG identifiers for the human genes that were included in this analysis, and related KO groups, if available (starting with hsa or K, respectively), are listed below. Keap1 - hsa:9817/K10456. Nrf2 (NFE2L2) – hsa:4780/K05638 (orthologous genes from vertebrates and *Caenorhabditis elegans*)/K09041 (contains cnc, a known functional ortholog of Nrf2 from Drosophila melanogaster, and related orthologs from several other invertebrates). Nrf1 (NFE2L1) – hsa:4779/K09040 (this orthology group contains also more distantly related paralogous Nrf3 (NFE2L3) genes, which lack ETGE box and Keap1 binding ability, and were thus excluded from the analysis). MCM3 – hsa:4172/K02541. PALB2 – hsa:79728/K10897. CHD6 – hsa:84181/K14436. OTUD1 – hsa:220213/K13716. PGAM5 – hsa:192111/K15637. DFFA – hsa:1676/K02310. DPP3 – hsa:10072/K01277. AMER1 – hsa:139285/K19407. SLK – hsa:9748/K08836. TRIM37 – hsa:4591/K10608. CUL2 – hsa:8453/K03870. IKBKB - K07209/hsa:3551. SQSTM1 - hsa:8878/K14381. MAD2L1 – hsa:4085/K02537. ATP6V0A2 - hsa:23545/K02154 (this large KO group contains several paralogs of the gene, from which the closer orthologs related to ATP6V0A2 were identified with the help of the KEGG SSDB list of the human ATP6V0A2 gene). WDR1 - hsa:9948. C8orf47 – hsa:203111. FAM117B – hsa:150864. FAM129B – hsa:64855. TSC22D4 – hsa:81628. LAMA1 – hsa:284217/K05637. BRCA2 (FANCD1) - hsa:675/K08775. EEF2 - hsa:1938/K03234. CDK20 (CCRK) - hsa:23552/K08817. Protein sequences for the identified orthologs were retrieved using DBGET/KEGG integrated database retrieval system queries and multiple sequence homology alignment of retrieved sequences for any given gene was carried out using Clustal W option integrated to the KEGG query results page.

### Yeast strains and methods

All *Saccharomyces cerevisiae* strains were congenic with W303 and are listed in the Supplementary Table S1. All mutants of the *MCM3* were created by PCR, introducing the alternate sequences into oligonucleotide primers. AluI site was designed into the sequence of *mcm3*-*GAGA* mutant and XhoI site was used to replace sequences in *mcm3* H2I deletion mutants for easier detection of mutated alleles. In latter constructs, the XhoI site encodes two amino acids (Leu-Glu) that replace the original sequence. Mutated *mcm3* alleles were linked to *natMX6* marker gene and transformed into diploid W303 to replace one copy of genomic *MCM3*. Diploid strains were sporulated and spores from tetrads were dissected on YPD (yeast extract-peptone-dextrose) plates. To identify haploid clones carrying a mutant *mcm3* allele, the dissected spores were replica-plated to YPD supplemented with 100 μg/ml of nourseothricin (NAT).

For the competitive yeast co-growth assay, the strains carrying WT MCM3 (AKY990), mcm3-GAGA (AKY1080) or mcm3-del449-454 (AKY1135) alleles were grown overnight in YPD media, diluted to density 2 × 106 cells/ml and grown further for three generations. Then cells were counted and co-growth cultures were inoculated into 50 ml YPD by mixing WT strain with each mutant in 1:1 ratio and grown for four days at 30 °C. To ensure continuous growth of strains, the co-cultures were diluted by 50,000 fold after every 24 hours. 1 ml samples from co-cultures (typical density 1–2 × 107 cells/ml) were collected in every 24 hours and total genomic DNA was prepared by LiOAc method^88^. 810 bp fragment from MCM3 locus was amplified by PCR and cut with AluI (cuts mcm3-GAGA allele to 460 bp +350 bp fragments) or XhoI (cuts mcm3-del449-454 allele to 460 bp +340 bp fragments). DNA fragments were separated in 1% agarose gel and stained with ethidium bromide.

For the yeast plasmid maintenance assay, the strains carrying WT MCM3 (AKY990), mcm3-GAGA (AKY1080) or mcm3-del449-454 (AKY1135) alleles were transformed with pRS416 containing a centromere (CEN6), single replication origin (ARS4) and URA3 selection marker. Colonies from SC–ura plates were inoculated into 25 ml YPD and grown overnight. Next day cells were inoculated into 50 ml YPD and grown for additional four days at 30 °C. To ensure continuous growth of strains, the cells were diluted by 50,000 fold after every 24 hours. Every day approximately 400 cells were plated onto YPD and SC-ura plates and incubated at 30 °C for three days. Colonies from both plates were counted and the percentage of plasmid-carrying cells was calculated.

### Cell transfection and subcellular fractionation

cDNAs encoding for human WT Keap1, R380A/R415A double mutant Keap1, WT MCM3, or ETGE > GAGA mutant MCM3 were PCR cloned into the pQM expression vector (Quattromed, Estonia). All expressed proteins contained N-terminal FLAG affinity tag, which was additionally followed by MBP tag in MCM3 proteins. Human U2OS osteosarcoma cells (American Type Culture Collection, HTB-96) were grown in Iscove’s modified Dulbecco’s medium (IMDM) supplemented with 10% fetal calf serum (FCS) and transfected with plasmid DNA using GeneJammer transfection reagent and protocol provided by manufacturer (Agilent Genomics). Cells were harvested 48 hours after transfection.

In subcellular fractionation experiments, the cells were harvested by trypsin treatment and 1/5^th^ of the cells was lysed in the Laemmli sample buffer for the total protein analysis; the rest were fractionated using Subcellular Protein Fractionation Kit and respective protocol from Thermo Fisher Scientific. Protein levels and subcellular distribution were analysed by Western blotting.

### MCM3 RNAi knock-down experiments

To knock-down MCM3 gene expression, two different siRNA oligonucleotides were used. siRNA #1-GCAUUGUCACUAAAUGUUCUCUAGU is based on the previous study^52^; siRNA #2-GCAGGUAUGACCAGUAUAA was selected as an overlapping high confidence hit from the analysis by three different siRNA design programs (Thermo Block-IT,GE Dharmacon siDesign, InvivoGen siRNA Wizard). Nonspecific control siRNA was purchased form Ambion (Silencer Select, cat nr 4390843). The siRNAs were transfected into U2OS cells at 50 nM concentration, using Lipofectamine RNAiMAX (Invitrogen) according to the manufacturer’s protocol. 48 h following the transfection, the cells were treated with tBHQ or DEM for 6 hrs, lysed in Laemmli sample buffer and analysed by Western blotting.

## Electronic supplementary material

Supplementary Figures
