# Supplementary material for: Keap1–MCM3 interaction is a potential coordinator of molecular machineries of antioxidant response and genomic DNA replication in metazoa
Source: Sci Rep. 2018 Aug 14;8:12136. doi: 10.1038/s41598-018-30562-y (PMC6092318; doi:10.1038/s41598-018-30562-y)
Supplement: Supplementary file 1 — Supplementary Figures [file 41598_2018_30562_MOESM1_ESM.pdf]

Keap1–MCM3 interaction is a potential coordinator of molecular machineries of antioxidant response and genomic DNA replication in metazoa

Nele Tamberg, Siret Tahk, Sandra Koit, Kersti Kristjuhan, Sergo Kasvandik, Arnold Kristjuhan, and Ivar Ilves

Supplementary information

- **Table S1.** Yeast strains used in this study
- **Figure S1.** Identification of MCM3 interaction partners from CHO-EBNALT85 cells
- **Figure S2.** Full-length images of immunoblots and Coomassie stained gels from Fig.1a-b.
- **Figure S3.** Structure comparison of the MCM2-7 and CMG complexes, and kelch domain of Keap1.
- **Figure S4.** Full-length images of immunoblots from Fig.2e-f.
- **Figure S5.** Full-length images of immunoblots from Fig.3a.
- **Figure S6.** Full-length images of immunoblots from Fig.4a.
- **Figure S7.** Molecular weight distribution of purified HA-MCM-BP, strep-Keap1, and MCM2-7 proteins
- **Figure S8.** Ectopic overexpression experiments with Keap1 and MCM3 in human U2OS cells
- **References.** References to supplementary figure legends

Table S1. Yeast strains used in this study

Strain	Genotype
AKY990	W303, MAT a, *ura3 leu2-3,112 his3-11,15 trp1-1 ade2-1 can1-100 lys2 mcm3::natMX6-MCM3*
AKY1080	W303, MAT a, *ura3 leu2-3,112 his3-11,15 trp1-1 ade2-1 can1-100 lys2 mcm3::natMX6-mcm3-GAGA*
AKY1135	W303, MAT a, *ura3 leu2-3,112 his3-11,15 trp1-1 ade2-1 can1-100 lys2 mcm3::natMX6-mcm3-del449-454*
AKY987	W303, diploid, *ura3 leu2-3,112 his3-11,15 trp1-1 ade2-1 can1-100 lys2* MCM3/*mcm3::natMX6-MCM3*
AKY1079	W303, diploid, *ura3 leu2-3,112 his3-11,15 trp1-1 ade2-1 can1-100 lys2* MCM3/*mcm3::natMX6-mcm3-GAGA*
AKY966	W303, diploid, *ura3 leu2-3,112 his3-11,15 trp1-1 ade2-1 can1-100 lys2* MCM3/*mcm3::natMX6-mcm3-del444-459*
AKY1132	W303, diploid, *ura3 leu2-3,112 his3-11,15 trp1-1 ade2-1 can1-100 lys2* MCM3/*mcm3::natMX6-mcm3-del444-448*
AKY1133	W303, diploid, *ura3 leu2-3,112 his3-11,15 trp1-1 ade2-1 can1-100 lys2* MCM3/*mcm3::natMX6-mcm3-del449-454*
AKY1134	W303, diploid, *ura3 leu2-3,112 his3-11,15 trp1-1 ade2-1 can1-100 lys2* MCM3/*mcm3::natMX6-mcm3-del455-459*

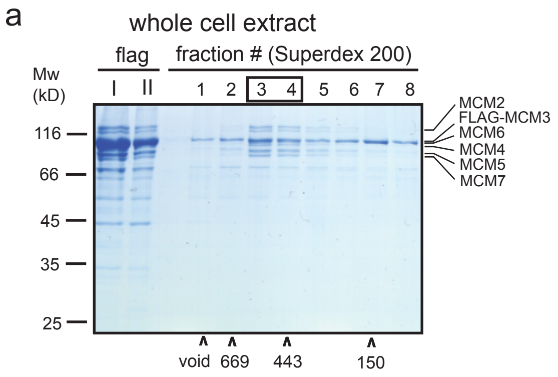

b

Name	Accession	Mw	Peptides 3+4	Coverage % 3+4	Intensity 3+4
**MCM3**	P25206	91.5	72	80.8	7.7E+10
**MCM5**	XP_007628260	82.3	64	77.7	2.5E+10
**MCM6**	XP_003497763	93.0	62	67	2.4E+10
**MCM4**	XP_007638104	96.5	59	70	2.5E+10
**MCM2**	XP_007639150	102.2	59	64	2.4E+10
**MCM7**	XP_007623926	81.0	45	67.9	2.6E+10
**Keap1**	XP_007624296	69.6	31	59.3	1.5E+09
**MCMBP**	XP_007623047	72.0	22	44.3	8.5E+08

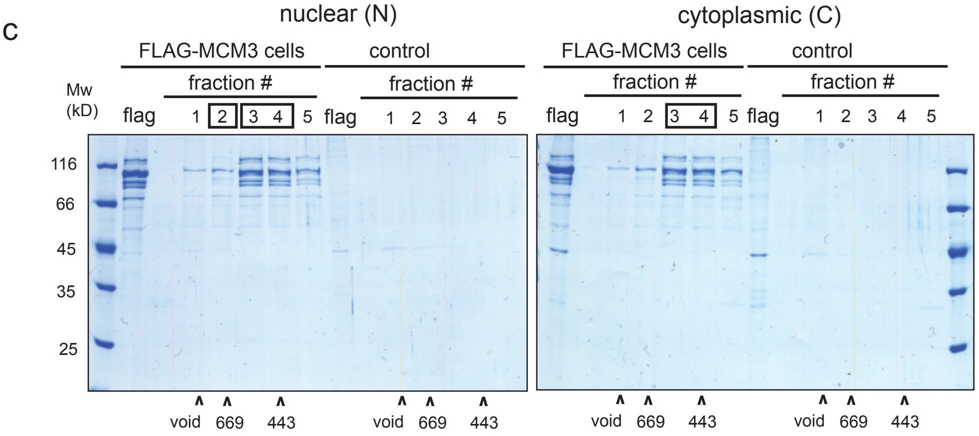

d

Name	Accession	Mw	Peptides N2	N3+4	C3+4	Seq. coverage % N2	N3+4	C3+4	Intensity N2	N3+4	C3+4
**MCM3**	P25206	91.5	74	81	78	82.9	84.5	82.6	4.5E+11	3.3E+11	3.3E+11
**MCM5**	XP_007628260	82.3	41	68	66	64.9	79	77.9	1.3E+10	1.2E+11	1.1E+11
**MCM4**	XP_007638104	96.5	45	67	69	59.9	74.5	75.4	2.3E+10	1.8E+11	1.3E+11
**MCM6**	XP_003497763	93.0	57	67	67	65.4	74.4	71.6	4.9E+10	1.6E+11	1.4E+11
**MCM7**	XP_007623926	81.0	40	52	47	65.2	74.4	70.2	1.8E+10	1.6E+11	1.3E+11
**MCM2**	XP_007639150	102.2	53	65	60	60.1	72.3	67.6	2.3E+10	1.5E+11	1.2E+11
**MCMBP**	XP_007623047	72.0	18	25	28	38.8	50.5	55.5	1.1E+09	2.2E+09	3.5E+09
**Keap1**	XP_007624296	69.6	25	26	30	49.2	49.4	59	2.1E+09	9.2E+08	2.6E+09

Figure S1

Identification of MCM3 interaction partners from CHO-EBNALT85 cells

(a) Coomassie stained SDS-PAGE gel with the FLAG immunoprecipitated MCM3 complexes (‘flag’ I and II) and fractions from the subsequent Superdex 200 size exclusion chromatography step carried out with the material from the first FLAG eluted fraction. Co-elution of molecular weight markers is shown at the bottom, and the fractions that were pooled for mass spectrometry (MS) analysis are marked with boxes both here and in panel C. Proteins were immunoprecipitated from the total extract of CHO-EBNALT85 cells expressing FLAG tagged MCM3.

(b) Table showing the most abundant specific partners of MCM3 from pooled fractions of 3 and 4 in panel ‘a’ according to the MS analysis.

(c) Coomassie stained gel of the analogous experiment that was carried out separately from the nuclear and cytoplasmic fractions of the CHO-EBNALT85 cells expressing FLAG-MCM3. The data shown here and in Fig. 1b are from two independent experiments. Control lanes show the corresponding fractions from parallel control experiment with parent CHOEBNALT cells lacking the FLAG-MCM3 expression.

(d) Most abundant specific binding partners of MCM3 according to the MS analysis of the fractions indicated in panel ‘c’.

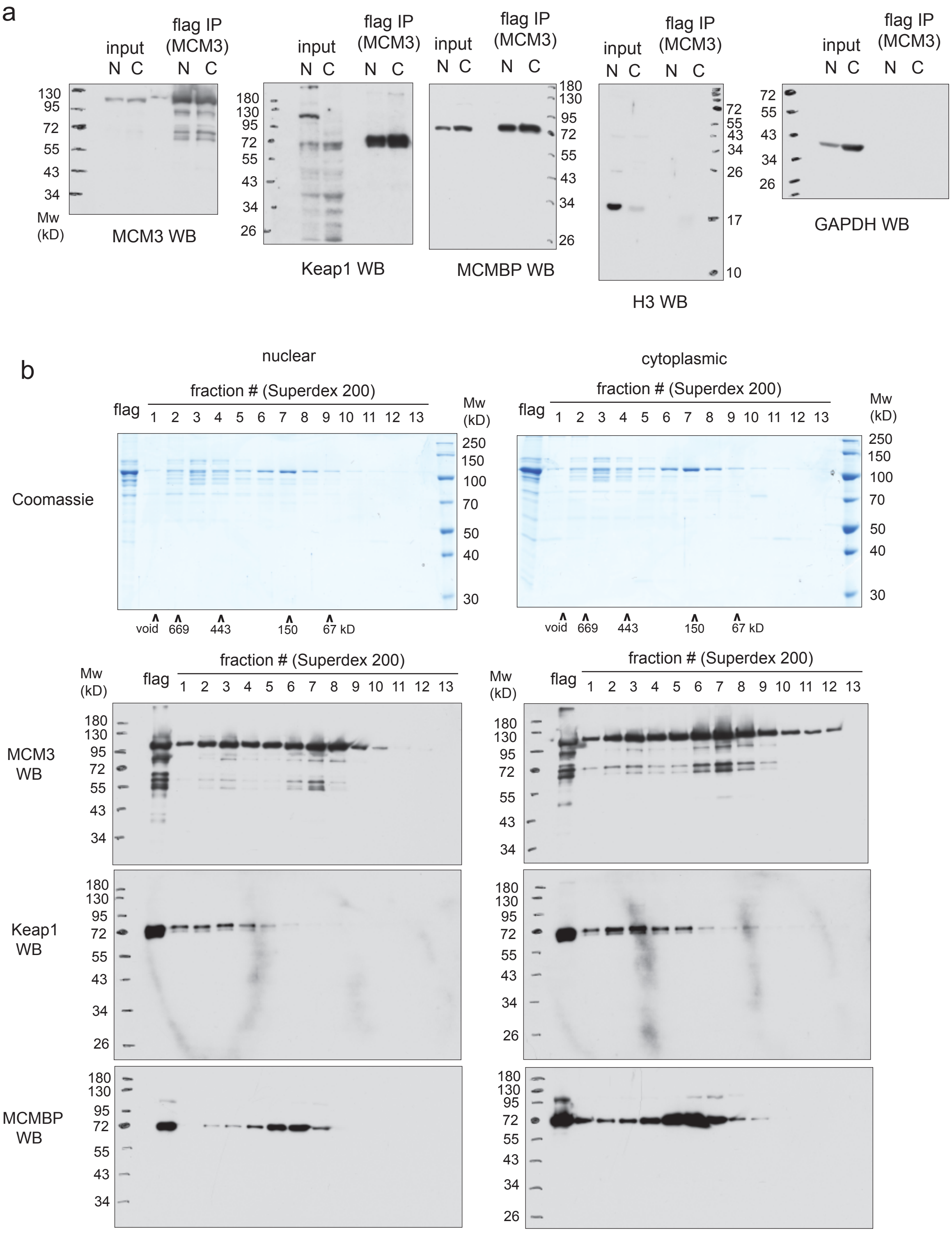

Figure S2

Full-length images of immunoblots and Coomassie stained gels from Fig.1a-b.

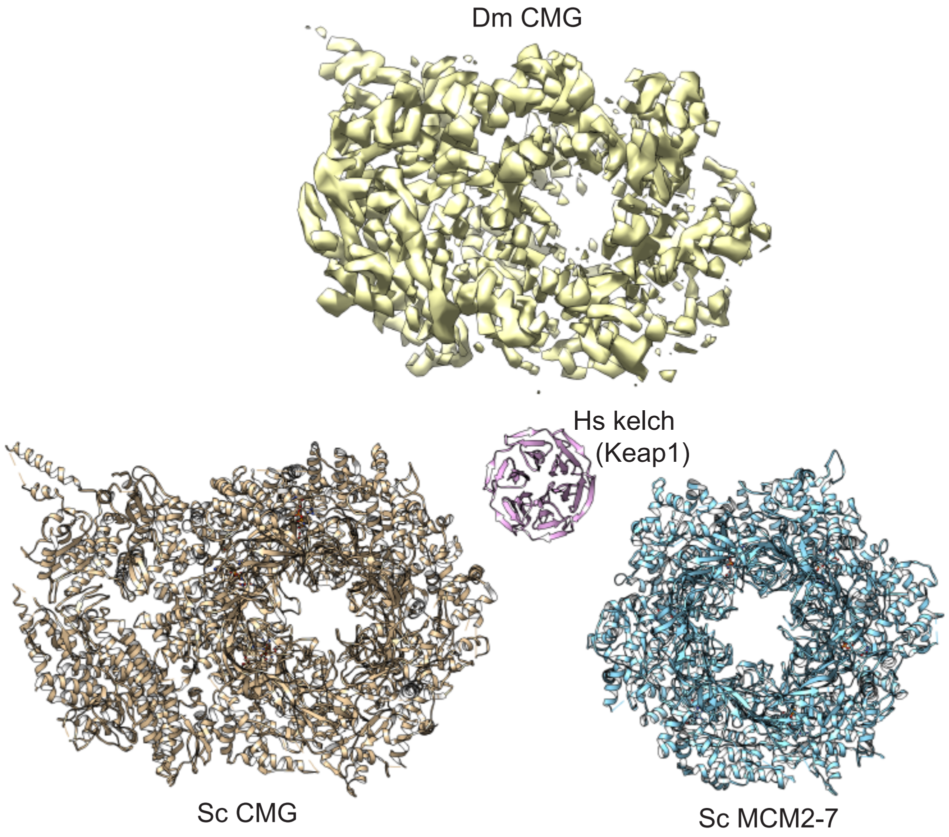

Figure S3

Structure comparison of MCM2-7 and CMG complexes, and kelch domain of Keap1.

Kelch domain of human Keap1 is shown in the middle (PDB accession code 2flu (Lo et al., 2006)), *Saccharomyces cerevisiae* MCM2-7 on the bottom right (PDB accession code 3JA8 (Li et al., 2015)), *S. cerevisiae* CMG on the bottom left (PDB accession code 5U8T (Georgescu et al., 2017)), and *Drosophila melanogaster* CMG on the top (EMDB accession code 3318 (Abid Ali et al., 2016)). Shown are the top views on the N-terminal tier side of the MCM2-7 and CMG complexes, and the bottom view from the side opposite to the DxETGE motif binding pocket of Kelch domain.

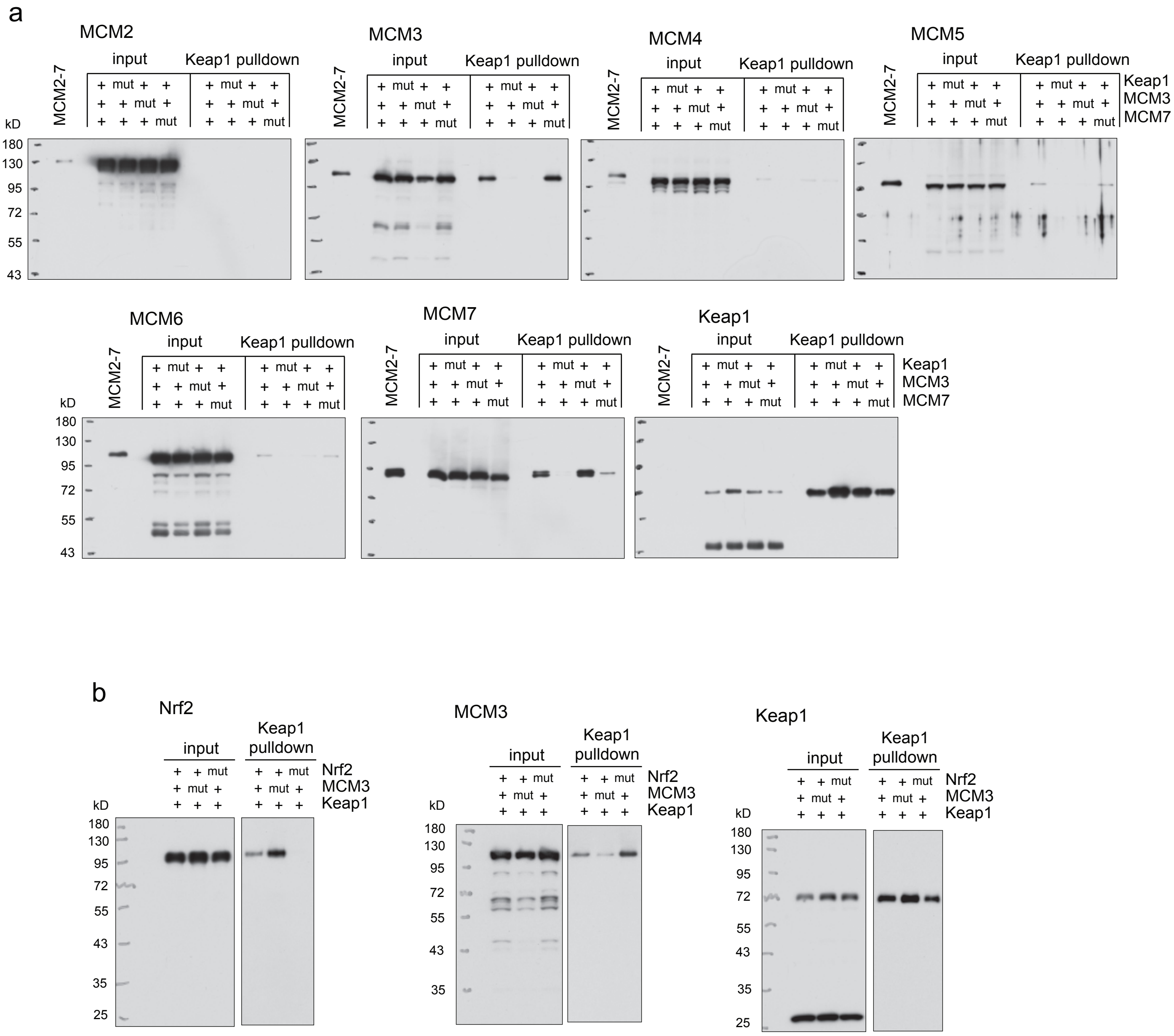

Figure S4

Full-length images of immunoblots from Fig.2e-f.

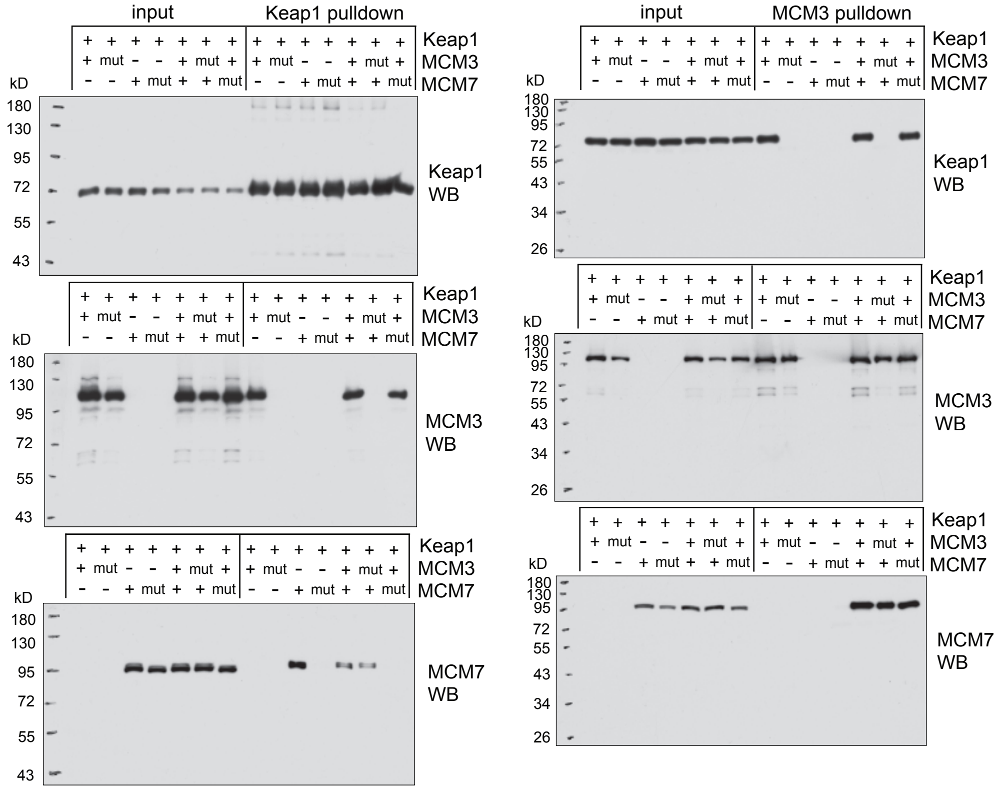

Figure S5

Full-length images of immunoblots from Fig.3a.

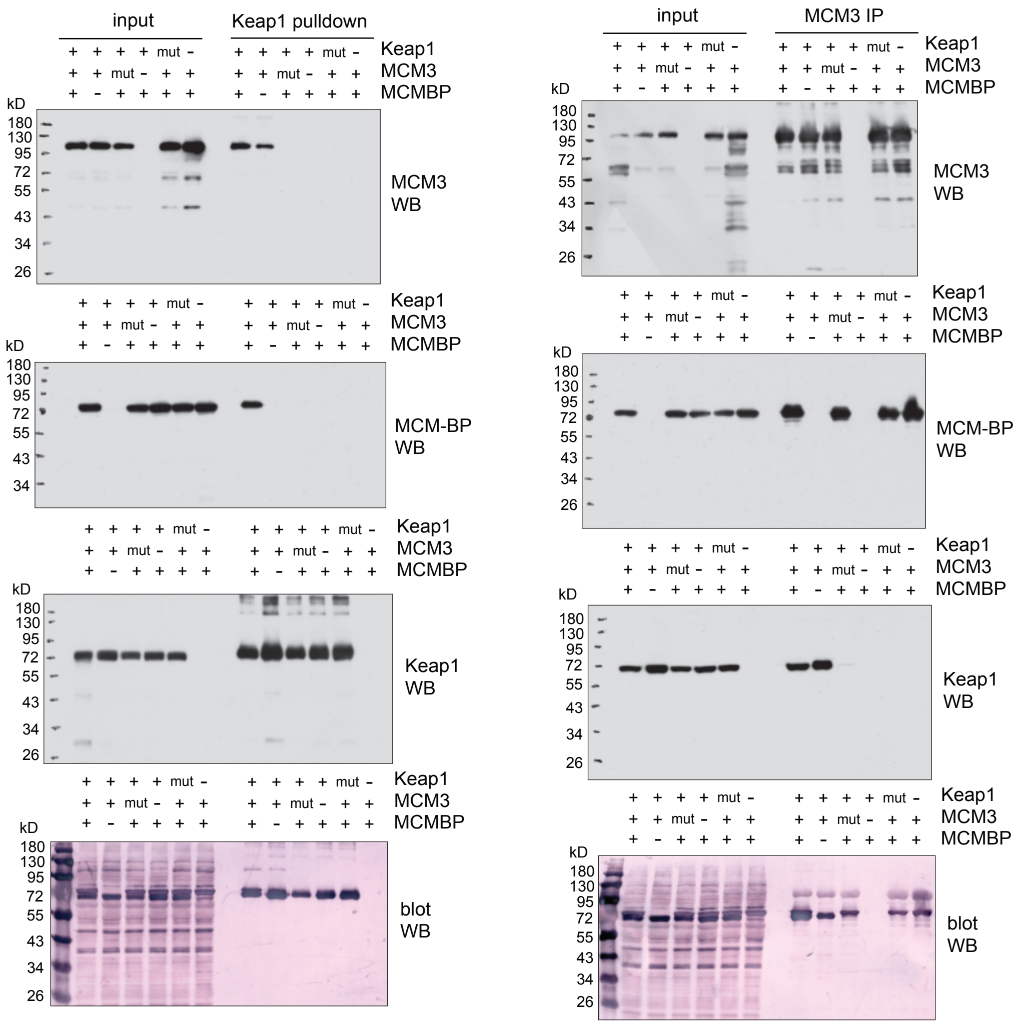

Figure S6

Full-length images of immunoblots from Fig.4a.

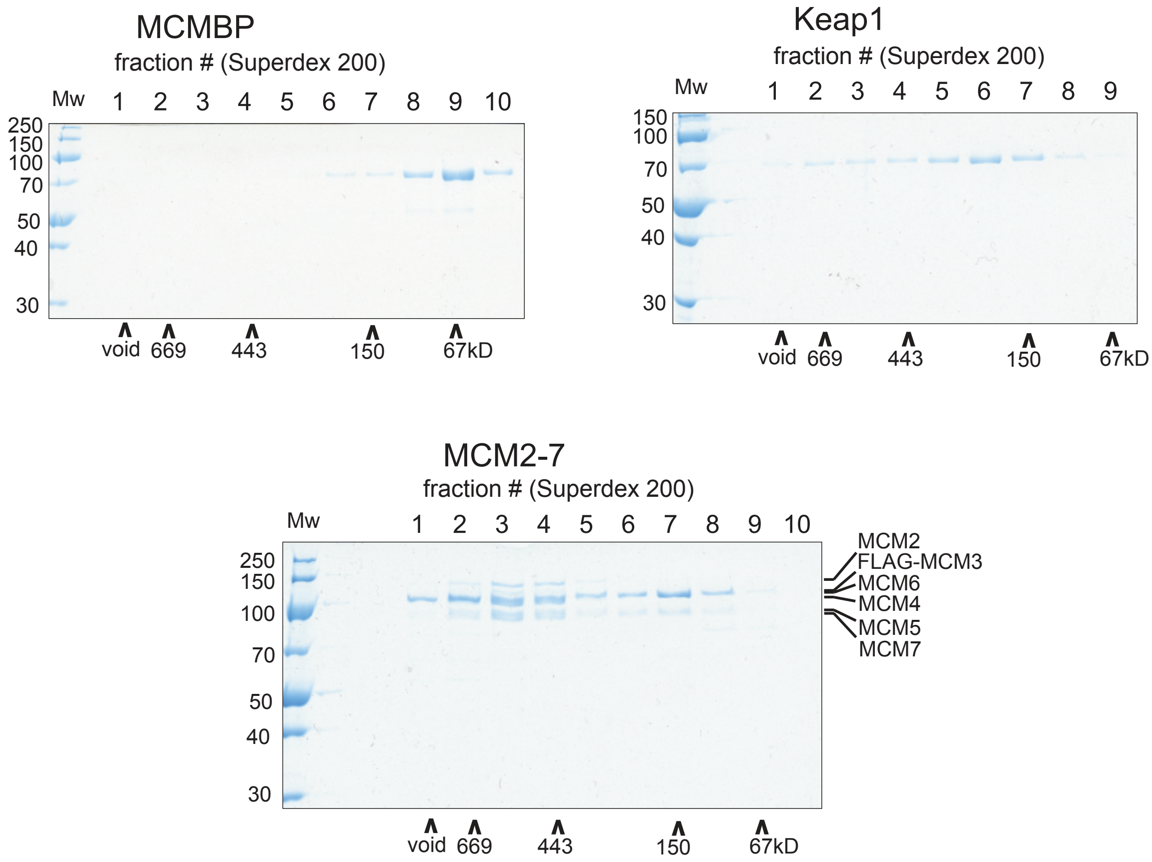

Figure S7

Molecular weight distribution of purified HA-MCM-BP, strep-Keap1, and MCM2-7 proteins

Coomassie brilliant blue stained SDS-PAGE protein gels showing the fractions of Superdex 200 size exclusion chromatography of mouse MCM2-7 complexes, MCM-BP, or Keap1. The material used in the chromatography step was affinity purified from the extracts of baculovirus infected Sf9 cells, using similar protocol and buffer conditions as in Fig. 4. MCM-BP and Keap1 were affinity purified with the help of N-terminal HA and strep affinity tags, respectively, and the MCM2-7 complexes were pulled down from the baculovirus co-infected cells with FLAG-tagged MCM3. Co-elution of molecular weight markers is shown at the bottom of respective fraction lanes. These data show efficient pulldown of heterohexameric MCM2-7 complex by MCM3 peaking in fraction #3, also confirm that the elution profiles of MCM-BP and Keap1 proteins alone are consistent with these proteins being preferably monomeric or dimeric in solution, respectively.

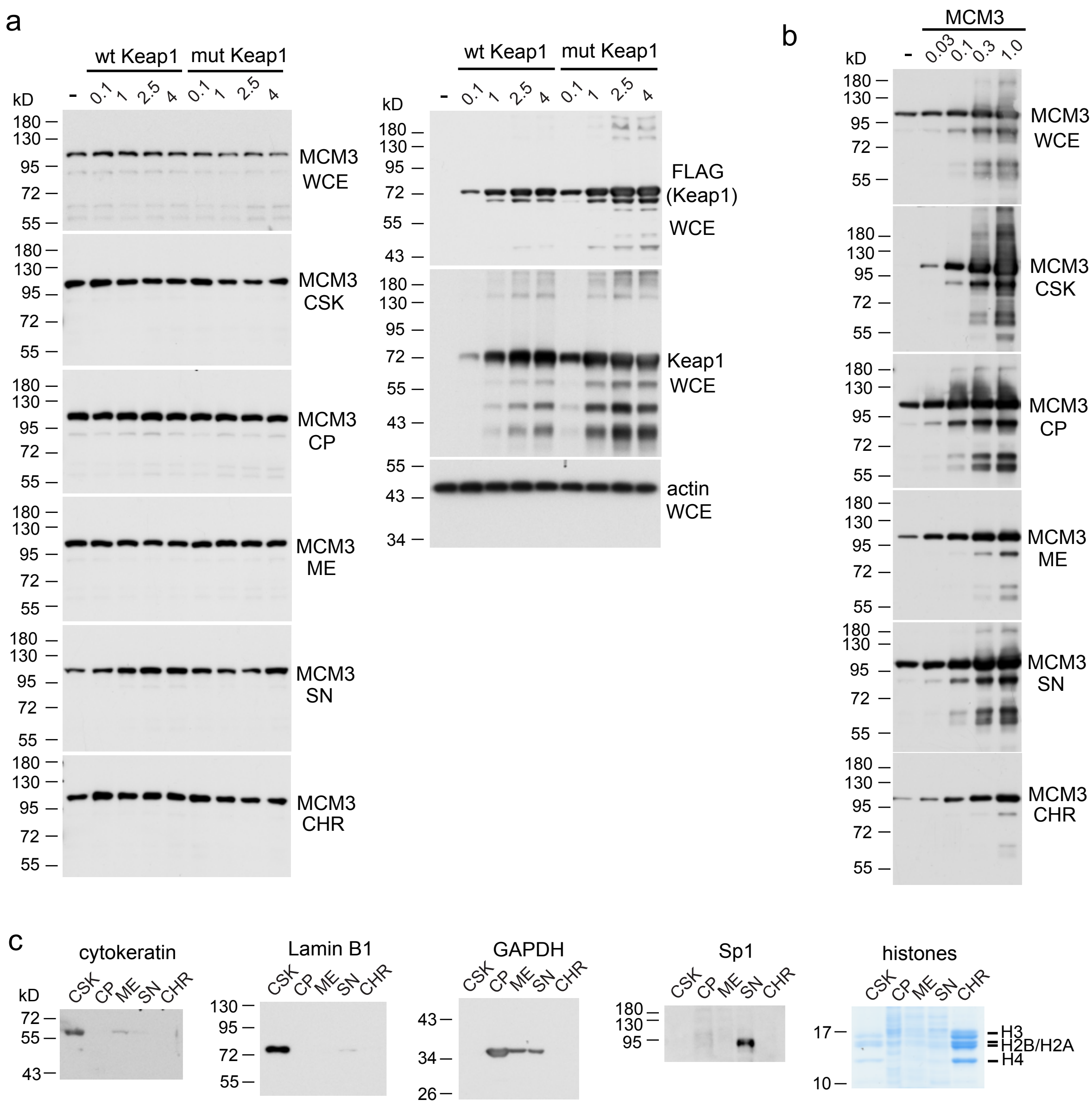

Figure S8

Ectopic overexpression experiments with Keap1 and MCM3 in human U2OS cells.

(a) Transient transfection experiments with rising amounts of plasmid constructs (µg of DNA as shown on top) expressing either WT or R380A/R415A mutant version of FLAG-Keap1. Shown are the Western blots with whole cell extracts (WCE) and following subcellular fractions: chromatin (CHR), soluble nuclear (SN), membrane (ME), soluble cytoplasmic (CP), and cytoskeleton enriched (CSK). Probed proteins are shown on right, actin serves as a loading control. The endogenous protein signal in the first control lane of the Keap1 WCE blot is undetectable at the shown sensitivity, indicative of strong ectopic overexpression of Keap1 protein in the transfected cells relative to the endogenous levels. Estimated transfection efficiencies of U2OS cells in our experiments were >50-60%.

(b) Transient transfection experiments with rising amounts of MCM3 expression vector (µg of DNA as shown on top). Here, the western blots show the amount of total MCM3 protein in the whole cell extracts and subcellular fractions of transfected cells, as the ectopically expressed MCM3 protein moves on top of the endogenously expressed protein. All the blots were developed together and have the same exposure time; the ratio of loaded material is 1xCSK : 2xCP : 2xME : 1.3xSN : 1.7xCHR.

(c) Western blots showing successful enrichment of marker proteins with the used fractionation protocol. Coomassie stained gel on right shows preferential extraction of core histones in chromatin fraction.

References

Abid Ali, F., Renault, L., Gannon, J., Gahlon, H.L., Kotecha, A., Zhou, J.C., Rueda, D., and Costa, A. (2016). Cryo-EM structures of the eukaryotic replicative helicase bound to a translocation substrate. *Nat. Commun.* **7**, 10708.

Georgescu, R., Yuan, Z., Bai, L., de Luna Almeida Santos, R., Sun, J., Zhang, D., Yurieva, O., Li, H., and O'Donnell, M.E. (2017). Structure of eukaryotic CMG helicase at a replication fork and implications to replisome architecture and origin initiation. *Proc. Natl. Acad. Sci. U.S.A.* **114**, E697-E706.

Li, N., Zhai, Y., Zhang, Y., Li, W., Yang, M., Lei, J., Tye, B.K., and Gao, N. (2015). Structure of the eukaryotic MCM complex at 3.8 A. *Nature* **524**, 186-191.

Lo, S.C., Li, X., Henzl, M.T., Beamer, L.J., and Hannink, M. (2006). Structure of the Keap1:Nrf2 interface provides mechanistic insight into Nrf2 signaling. *EMBO J.* **25**, 3605-3617.

Padmanabhan B, Tong KI, Ohta T, Nakamura Y, Scharlock M, et al. (2006) Structural basis for defects of Keap1 activity provoked by its point mutations in lung cancer. *Mol. Cell* **21**: 689-700.
